# *In vitro* co-culture of *Clostridium scindens* with primary human colonic epithelium protects the epithelium against *Staphylococcus aureus*

**DOI:** 10.3389/fbioe.2024.1382389

**Published:** 2024-04-12

**Authors:** Hao Wang, Raehyun Kim, Yuli Wang, Kathleen L. Furtado, Christopher E. Sims, Rita Tamayo, Nancy L. Allbritton

**Affiliations:** ^1^ Department of Bioengineering, University of Washington, Seattle, WA, United States; ^2^ Department of Biological and Chemical Engineering, Hongik University, Sejong, Republic of Korea; ^3^ Department of Microbiology and Immunology, University of North Carolina, Chapel Hill, NC, United States; ^4^ Department of Medicine/Division of Rheumatology, University of Washington, Seattle, WA, United States

**Keywords:** intestine, gut microbiome, multi-species culture, oxygen gradient, bile acid, enterotoxin, interactions

## Abstract

A complex and dynamic network of interactions exists between human gastrointestinal epithelium and intestinal microbiota. Therefore, comprehending intestinal microbe-epithelial cell interactions is critical for the understanding and treatment of intestinal diseases. Primary human colonic epithelial cells derived from a healthy human donor were co-cultured with *Clostridium scindens* (*C. scindens*), a probiotic obligate anaerobe; *Staphylococcus aureus* (*S. aureus*), a facultative anaerobe and intestinal pathogen; or both bacterial species in tandem. The co-culture hanging basket platform used for these experiments possessed walls of controlled oxygen (O_2_) permeability to support the formation of an O_2_ gradient across the intestinal epithelium using cellular O_2_ consumption, resulting in an anaerobic luminal and aerobic basal compartment. Both the colonic epithelial cells and *C. scindens* remained viable over 48 h during co-culture. In contrast, co-culture with *S. aureus* elicited significant damage to colonic epithelial cells within 24 h. To explore the influence of the intestinal pathogen on the epithelium in the presence of the probiotic bacteria, colonic epithelial cells were inoculated sequentially with the two bacterial species. Under these conditions, *C. scindens* was capable of repressing the production of *S. aureus* enterotoxin. Surprisingly, although *C. scindens* converted cholic acid to secondary bile acids in the luminal medium, the growth of *S. aureus* was not significantly inhibited. Nevertheless, this combination of probiotic and pathogenic bacteria was found to benefit the survival of the colonic epithelial cells compared with co-culture of the epithelial cells with *S. aureus* alone. This platform thus provides an easy-to-use and low-cost tool to study the interaction between intestinal bacteria and colonic cells *in vitro* to better understand the interplay of intestinal microbiota with human colonic epithelium.

## 1 Introduction

The colon receives partially digested food from the small intestine and absorbs water and residual nutrients, subsequently transporting indigestible waste in the form of feces for elimination (Azzouz and Sharma, 2022). The colon is lined with a monolayer of epithelial cells that form a barrier between luminal contents and the host while maintaining an anaerobic environment within the lumen (Kim et al., 2019). Additionally, the colon has less fluid and the lowest average flow rate of the intestinal system (Harris and Scolapio, 2019). Based on these features, the colon provides an ideal growth environment for a complex microbial community that includes obligate and facultative anaerobic species and whose composition and metabolism are influenced by diet and host factors. In turn, this microbial community contributes to human metabolism through fermentation of undigested fiber (Clarke et al., 2014), production of vitamins (Guarner and Malagelada, 2003; Azzouz and Sharma, 2022), absorption of minerals (Guarner and Malagelada, 2003), and transformation of bile acids (Ridlon et al., 2014). Commensal microbiota also plays an essential role in the defense against pathogens (Cheng et al., 2020; Khan et al., 2021). Known mechanisms in this defense include direct pathogen inhibition by metabolic products of commensal microbes (Rooks and Garrett, 2016), outcompeting pathogens for nutrients and other resources that limit pathogen growth (Stubbendieck and Straight, 2016), formation of a microbial barrier in the mucosal layer (Sommer and Bäckhed, 2013), and directing the immune system towards elimination of pathogens (Baruch et al., 2021; Davar et al., 2021). Commensal microbes also interact with and regulate the colonic epithelium itself, influencing cell turnover, promoting epithelial restitution, and reorganization of tight junctions (Yu et al., 2012). In contrast, pathogenic microbes have the potential to degrade the mucus barrier and invade the mucosal epithelium; these pathogens cause or are associated with various intestine-associated diseases, including gastroenteritis, inflammatory bowel disease (IBD), pancreatitis, and cancer (Shu et al., 2023). Therefore, understanding host-microbiota interactions is essential for the preservation of human health and may lead to effective approaches to mitigate intestinal diseases.

The interactions between host and intestinal microbiota are often studied using animal models. To limit or prevent intestinal microbe colonization, animals are either raised in a germ-free environment and/or exposed to antibiotic treatment to allow control of microflora during experiments (Sivan et al., 2015). The most common animal model is the mouse due primarily to accessibility and low maintenance cost (Nguyen et al., 2015; Li and Zhang, 2022). The porcine model has a more comparable colonic structure, hormone secretion, and gut microbiota composition to the human (Heinritz et al., 2013; Roura et al., 2016), which makes the pig a popular, albeit costly, animal model. Although animal models have been commonly used in gut microbiome research for decades, ethical and cost issues, their distinct genetics, intestinal structure, microbiota composition, and immune responses impose limitations on these models for understanding human/microbiome physiology and pathophysiology (Marrero et al., 2021). *In lieu* of animal models, *in vitro* fermentation systems using reactor chambers are cost-effective platforms for intestinal microbiota studies (Li and Zhang, 2022). These batch or continuous flow systems enable precise control of physiological parameters within the system, such as pH, temperature, retention time, composition of the media, and anaerobic conditions (Payne et al., 2012). Multistage fermentation models enable the simulation of the different luminal physiological environments from the stomach to the colon in the absence of intestinal epithelium (Verhoeckx et al., 2015). However, these systems are unsuitable for assessing interactions with the host gastrointestinal tissue. To address this limitation, viable intestinal segments from animals have been integrated into fluidic systems to assemble *ex vivo* models, such as the Ussing chamber and the everted gut sac model, although these systems have many challenges, including their size, operational complexity, and the absence of human intestinal epithelium (Clarke, 2009; Alam et al., 2012; Rahman et al., 2021).

To replicate the interaction between human intestine and gut microbiota, *in vitro* models combining microfluidic platforms and co-culture of primary human intestinal cells with gut bacteria have been developed. These systems have been used to create a more complex environment for intestinal microbiota by perfusing oxygenated and deoxygenated media in separate compartments to create an O_2_ gradient and fluidic shear stress, as well as to prevent bacterial overgrowth and to collect secreted molecules (Tovaglieri et al., 2019; Zhang et al., 2021b). Despite their potential, the complex, sterile flow systems and enclosed structure (with inaccessible compartments) present barriers in their use. Organoids embedded in Matrigel™ formed from differentiated gastrointestinal stem cells have been used to model an internal cavity representing the gut lumen, with live bacteria inoculated through microinjection (Bartfeld et al., 2015; Williamson et al., 2018). Shortcomings of these models are the difficulty of accessing the luminal compartment, an uncontrolled luminal O_2_ concentration, and overgrowth of the confined microbiota. To partially address these limitations, the polarity of organoids has been reversed by disrupting the polymerization of the ECM proteins to create “luminal-out” organoids embedded in media (Co et al., 2019). Luminal-out organoids can be transferred to media containing microbes to achieve co-culture experiments, but this organoid system lacks the ability to create the low-O_2_ concentration needed to support anaerobic bacteria and presents further challenges in controlling and sampling the embedded tissue mimic.

Hanging basket systems have been applied to investigate host-bacteria interactions (Roodsant et al., 2020; Sasaki et al., 2020; Zhang et al., 2021a). The double-sided design of this platform, with luminal and basal compartments separated by a monolayer of mammalian gastrointestinal epithelial cells, makes it feasible to mimic the luminal-epithelial structure of intestinal tissue and eases inoculating microbiota and sampling the luminal and basal environments (Dutton et al., 2019; Šuligoj et al., 2020). Recently, a hanging basket system with an oxygenated basal reservoir and deoxygenated luminal compartment was shown to form an O_2_ gradient across a primary human colonic epithelium; this system supported the human epithelial cells while enabling co-culture of obligate anaerobic bacteria in the luminal compartment (Kim et al., 2019; Kim et al., 2022). This platform does not require continuous perfusion of the media and allows routine sampling and replenishment of both luminal and basal environments when used in combination with an anaerobic chamber, making it attractive and accessible to a broad group of users. However, to date, co-culture of the human epithelium with only a single strain of bacteria for a short duration (24 h) has been demonstrated with this system. In the current study, we extend the lifetime of co-culture to 48 h with the commensal bacterium *Clostridium scindens* and with the pathogen *Staphylococcus aureus* when the bacteria are co-cultured in tandem. The goal of this experimental work is to determine whether a colonic commensal obligate anaerobe can be co-cultured in the presence of living primary human colonic epithelial cells with high viability and metabolic activity, and whether this co-culture affords protection to the colonic epithelium in the presence of pathogenic bacteria.

## 2 Materials and methods

### 2.1 Fabrication of an O_2_ gradient cassette with a supporting collagen matrix

The O_2_ gradient cassette was composed of a basal fluid reservoir, a customized hanging basket (similar to a Transwell insert) placed into the basal reservoir, and an O_2_-impermeable machined plug for insertion into the luminal portion of the hanging basket (Supplementary Figure S1). A conventional 12-well plate was used for the basal reservoirs. The design and fabrication of the hanging basket was similar to that described previously (Gunasekara et al., 2018; Kim et al., 2019). Briefly, the hanging basket was fabricated from polycarbonate using a CNC milling machine. Next, a porous polyester (PET) membrane (0.4 µm pores, Sterlitech Cat #1300016) was attached to the bottom of the hanging basket using double-sided medical grade tape (3M, Cat #1504XL). Five minutes of plasma treatment (Harrick Plasma, Cat #PDC-32G) improved adhesion of subsequently added collagen to the basket. The basket with 200 µL neutralized collagen (rat tail, type I, Corning, Cat #354236) solution (1 mg/mL) (Kim et al., 2019; Kim et al., 2022) was incubated for 1 h at 37°C and then submerged in 3 mL crosslinking solution added to the basal reservoir for 30 min at 25°C. The crosslinking solution was 0.6 M 1-Ethyl-3-(3-dimethylaminopropyl)-carbodiimide (EDC): 0.15 M N-hydroxysuccinimide (NHS): 0.1 M 2-(N-morpholino) ethanesulfonic acid (MES) buffer (pH 5.0) in water, (1:1:1 (vol). Later, the basket with partially crosslinked collagen matrix (1.1 cm^2^ of surface area and 2.2 mm of thickness (Gunasekara et al., 2018)) was incubated in deionized water for >18 h at 25°C to leach out residual crosslinking reagent followed by sterilization in 70% ethanol and was then air-dried in a biosafety cabinet. The basket with collagen matrix could be stored in PBS buffer at 4°C for 2–3 weeks before adding the human primary colon cells. The cells were cultured aerobically to form a confluent monolayer on the collagen/PET membrane scaffold submerged in a well of the 12-well plate (see below). For O_2_ gradient, an O_2_-impermeable plug fabricated from polycarbonate was threaded into the luminal portion of the basket. A soft Viton^®^ fluoroelastomer O-ring (McMaster Car, Cat #1284N114) was used with the plug to form an O_2_-impermeable seal. A port in the plug was used for insertion of an O_2_ probe, and this port was sealed with ethylene propylene diene monomer (EPDM) rubber caps (McMaster-Carr, Cat #6448K117) when an O_2_ sensor was not in place. The O_2_ probe in the luminal compartment was a needle O_2_ probe (PreSens, NTH-PSt7/Microx4, Germany). The tip of the probe was submerged into the luminal media to approximately 2 mm above the epithelial monolayer when O_2_ was measured.

### 2.2 Human primary colon epithelial cell culture

Primary human colonic epithelial stem cells from the transverse colon of a cadaveric donor (male, 23 years old, RRID: CVCL_ZR41 (https://web.expasy.org/cellosaurus/CVCL_ZR41)) were isolated, expanded, and maintained on a collagen scaffold in a 6-well plate in a stem cell media (SM, Supplementary Table S1) as described previously (Wang et al., 2017; Kim et al., 2019). Cells were used between passage 7 and 12. For each experiment, the human colonic stem cells were sub-cultured from a single well of the maintenance culture into 6 hanging baskets with collagen matrix prepared as described above. The human colonic cells were expanded on the collagen matrix in the hanging basket in expansion medium (EM, Supplementary Table S1 0.5 mL placed in the luminal compartment and 3 mL placed in the basal compartment) for 6 days with media exchange every other day (Kim et al., 2019; Kim et al., 2022). After a confluent monolayer was formed on day 7, both luminal and basal media were replaced by differentiation medium without antibiotics (DM-Ab, Supplementary Table S1 0.5 mL placed in the luminal compartment and 3 mL placed in the basal compartment). On day 8, the luminal medium was replaced with 0.5 mL 10% peptone-yeast-fructose media (PYF, Supplementary Table S2) in 1× PBS.

### 2.3 Bacterial culture

*C. scindens* (ATCC 35704) was purchased from ATCC and propagated within an anaerobic chamber (Coy Laboratory Products). The bacteria were cultured in PYF medium (peptone 10 g/L, yeast extract 10 g/L, fructose 10 g/L, resazurin 1 mg/L, L-cysteine hydrochloride 0.5 g/L, CaCl_2_·H2O 100 mg/L, MgSO_4_·7H_2_O 50 mg/L, K_2_PO_4_ 40 mg/L, KH_2_PO_4_ 40 mg/L, NaHCO_3_ 0.4 g/L, NaCl 80 mg/L, hemin (5 mg/L) and vitamin K (1 mg/L)). *C. scindens* was plated on PYF agar (PYF with 15 g/L agar) and cultured anaerobically at 37°C using a BD GasPak EZ pouch system (BD, Cat # 260683). Two days before each experiment a fresh subculture was generated from a single colony for co-culture experiments by culturing in the PYF medium for 48 h.

*S. aureus* strain Newman was kindly provided by Dr. Michelle Reniere at the University of Washington and cultured under aerobic conditions in brain-heart-infusion media (BHI, Research Products International, Cat #B11000) at 37°C. *S. aureus* was plated on BHI agar (BHI with 15 g/L agar), and a single colony was used to prepare a bacterial sample after 18 h of culture in BHI broth before co-culture experiments.

### 2.4 Co-culture experiments

After human colonic epithelial cells fully differentiated in the luminal compartment of the hanging baskets, the luminal medium was substituted with a deoxygenated medium consisting of 10% PYF in 1× PBS buffer. This co-culture medium was enriched with 200 µM cholic acid for bile acid metabolism. Simultaneously, the basal medium was refreshed with fresh DM-Ab media. For *C. scindens* co-culture, 5×10^3^ colony-forming unit (CFU) in total were added to the luminal media similar to that used previously for other *Clostridioides* (Kim et al., 2019). The sealed co-culture or control cultures without bacteria were incubated at 37°C for 48 h. Due to its rapid growth, a minimal number of *S. aureus* (5 × 10^2^ CFU in total) was added to specified samples after 24 h of co-culture with *C. scindens*. The samples with added *S. aureus* were then incubated at 37°C for an additional 24 h. The deoxygenated luminal media was 10% PYF in 1 x PBS buffer for both *C. scindens* and *S. aureus*. All inoculations were performed in an anaerobic chamber.

### 2.5 Measurement of epithelial cell death

To assess the viability of colonic epithelial cells cultured in the O_2_ gradient cassette, DM-Ab medium (3 mL) supplemented with 10 μg/mL Hoechst 33342 (Thermo Fisher, Cat #H1399) was added to the basal compartment and then the cells were incubated at 37°C for 1 h. After washing with PBS, 2 μg/mL propidium iodide (PI, Thermo Fisher, Cat # P3566) in PBS buffer (3 mL) was added to the basal compartment, and then incubated at 37°C for 20 min. Hoechst 33342 was used to label DNA in all epithelial cells, and PI was used to label the DNA of dead epithelial cells. To measure apoptosis, a dead cell apoptosis kit with Annexin V (Thermo Fisher, Cat #V13242) was used to assess the presence of phosphatidylserine on the plasma membrane of cells. The working solution of the kit contained 1 μg/mL Annexin V, 1 μg/mL PI, 1.25 mM HEPES, 7 mM NaCl, 0.05 mM EDTA, and 0.005% bovine serum albumin (BSA) in 1× binding buffer (10 mM HEPES, 140 mM NaCl, and 2.5 mM CaCl_2_). 500 μL of the working solution was used for luminal staining at 25°C for 15 min, then the reaction was terminated by mixing with 2 mL binding buffer and kept on ice before imaging.

Prior to microscopic imaging, each collagen scaffold with attached cells was carefully cut from the hanging basket, inverted, and placed onto a #1 coverslip. A confocal fluorescence microscope (Olympus, Fluoview 3,000, Japan) was used to measure the fluorescence of the epithelial cells. The entire epithelial monolayer (approximately 1.1 cm^2^ surface area) was imaged as a 4 × 4 array with a 4× (0.16 N.A.) objective. The following fluorescence excitation/emission wavelengths were used- Hoechst 33342: Ex 405 nm, Em 430–470 nm; PI: Ex 561 nm, Em 610–670 nm; Annexin V: Ex 488 nm, Em 530–575 nm. All 16 images of each channel were stitched together to create an image of the entire well.

CellProfiler v4.0 was used to identify and calculate the image area with fluorescence intensity above an empirically set threshold to quantify the area of the epithelial monolayer positive for each of the stains. The processing protocol was modified from a previously reported method (Hinman et al., 2021). The stitched image at each emission wavelength was flat-field corrected using a Gaussian-smoothed illumination correction function (1000-pixel filter size). The corrected image was converted to binary using 2-class Otsu threshold selection. Then the area of the culture above the fluorescence threshold was calculated. The normalized PI + area was calculated as PI + area/Hoechst 33342+ area and was used as a metric for dead cells. The normalized Annexin V+ area was calculated as Annexin + area/Hoechst 33342+ area and used as a metric for apoptotic cells.

### 2.6 Immunofluorescence measurement of the tight junction marker ZO-1

To determine whether tight junctions among the epithelial cells were disrupted in the presence of the bacteria, methanol (4°C) was added as a fixative into the luminal compartment, and the sample was placed at −20°C for 1 h. The fixed cells were then washed in PBS and blocked with 3% BSA in PBS for 1 h at 25°C. Next, 200 µL ZO-1 primary antibody (2 μg/mL, Proteintech, Cat #21773-1-AP, RRID:AB_10733242) was added to the luminal compartment and incubated with the cells at 4°C for 18 h. The sample was then washed 3 times with IF wash buffer (0.1% BSA, 0.2% Triton-X 100, 0.05% Tween 20 in 1× PBS) and then incubated with 500 µL secondary antibody conjugated with Alexa Fluor 647 (1 μg/mL, Thermo Fisher Scientific, Cat #A27040, RRID:AB_2536101) containing 10 μg/mL Hoechst 33342 for 1 h at 25°C. The sample was then washed twice with PBS buffer. Prior to confocal microscopy, each collagen scaffold with attached cells was carefully cut from the hanging basket, inverted, and placed on a #1 coverslip. Fluorescence was then quantified using a 30× objective oil lens (1.05 N.A.). Fluorescence was measured using the following parameters- ZO-1: Ex 640 nm, Em 650–750 nm; Hoechst: Ex 405 nm, Em 430–470 nm. Five different locations (top, bottom, right, left, center) of each sample were imaged. The images were processed and analyzed within CellProfiler v4.0 following a previous protocol (Hinman et al., 2021).

### 2.7 Measurement of bacterial cells

The luminal contents (media + collagen scaffold) were collected and mixed in a tube with 3 mL 500 U/mL collagenase in PBS, followed by 30 min incubation at 25°C under anaerobic conditions. Next, the suspension was vortexed for 2 min, and then plated on PYF (for *C. scindens*) or BHI (for *S. aureus*) agar. The CFU on the agar plates after 24–48 h incubation anaerobically (for *C. scindens*) or aerobically (for *S. aureus*) was counted and used as a measure of the viability of the bacterial cells.

To visualize the bacterial cells while still present on the epithelial monolayer, 200 µL 1 μM SYTO 9 (Thermo Fisher, Cat #S34854) in PBS was added to the luminal compartment for 20 min. Epithelial cells were visualized by addition of Hoechst 33342 (10 μg/mL, PBS) followed by incubation for 1 h. The collagen scaffold with attached cells was carefully cut from the hanging basket, inverted, and placed on a #1 coverslip. Both bacterial and epithelial cells were imaged by confocal fluorescence microscopy using a 60× oil objective lens (1.3 N.A.) under the following parameters- Hoechst 33342: Ex 405 nm, Em 430–470 nm; SYTO9: Ex 488 nm, Em 500–520 nm.

### 2.8 ELISA assays of secreted cytokines

The IL-8 concentration in the basal media was measured using an interleukin 8 (IL-8) ELISA kit (Thermo Fisher Scientific, Cat #88-8086-22) following the manufacturer’s protocol. The basal media were diluted at 1:10 (control) or 1:20 (co-culture) so that the measured signals fit in the range of the standard curve provided with the kit. The MCP-1 concentration in the basal media was measured using a monocyte chemoattractant protein-1 (MCP-1) ELISA kit (Thermo Fisher Scientific, Cat #88–7399-88) following the manufacturer’s protocol.

### 2.9 Measurement of bile acid in epithelial-*Clostridium scindens* co-culture

After 48 h of co-culture in the presence of bile acid, both the luminal contents (media + collagen matrix) and basal media were collected separately. The luminal contents were degraded by collagenase as described above and the supernatant collected after centrifugation (10 min, 3,000×G). The luminal supernatant and basal media were assayed for bile acids by liquid chromatography-tandem mass spectrometry (LC-MS/MS) analysis as described previously (Ginos et al., 2018; Navarro et al., 2020) at the University of Washington Mitochondria and Metabolism Center.

As a control, cholic or deoxycholic acid was added to *S. aureus* cultured in 96-well plates. Diluted *S. aureus* (1/10^6^) was added to wells containing 200 µL 10% PYF (in 1× PBS) medium supplemented by 25–200 µM cholic acid or deoxycholic acid and incubated anaerobically. After 24 h at 37°C, the optical density (O.D.) at 600 nm of the medium in each well was measured.

### 2.10 Measurement of total enterotoxin produced by *Staphylococcus aureus*

The luminal contents (media + collagen scaffold) were collected and mixed with collagenase as described above. The supernatant was collected after centrifugation. The concentration of *S. aureus* enterotoxin in the supernatant was measured using a sandwich enzyme immunoassay kit (RIDASCREEN^®^ SET Total, R-Biopharm AG, German) following the manufacturer’s protocol. The O.D. of each sample was measured and an O.D. of 0.15 greater than that of the negative control value was set as a threshold for a positive sample. The concentration of enterotoxin was represented as relative O.D., *i.e.*, normalized to the positive control mean (set equal to 100%).

### 2.11 Statistical analyses

Origin 9 was used for statistical analyses of the data. All *p* values were calculated by ordinary one-way ANOVA. A significance level of 0.05 was used, and any *p*-value below this threshold was considered statistically significant. (*: *p* < 0.05, **: *p* < 0.01, ***: *p* < 0.001). In this manuscript, we define a “technical replicate” as being separate experiments on different days but using the cells of a single donor.

## 3 Results

### 3.1 Experimental overview and design

*C. scindens* is a bacterium often found in the human colon (White et al., 1980; MORRIS et al., 1985). *C. scindens* is remarkable for its 7-dehydroxylation activity that transforms primary bile acids (*e.g.*, cholic acid) to secondary bile acids (deoxycholic acid and lithocholic acid). Secondary bile acids are reported to inhibit the growth of intestinal pathogens, such as *S. aureus*, supporting a role for *C. scindens* as a probiotic species enhancing resistance to gastrointestinal infection (Buffie et al., 2015). *S. aureus* is a common cause of gastrointestinal illness after consumption of *S. aureus*-contaminated food (CDC, 2018). The toxicity of *S. aureus* is derived from enterotoxins that increase the permeability of the epithelial barrier and initiate epithelial cell death (Moretó and Pérez-Bosque, 2009; Šuligoj et al., 2020). The overall goal of this study was to co-culture these two bacterial species with primary human gastrointestinal epithelial cells to demonstrate the utility of multi-species gut-on-chip models for intestinal microbiota research and the study of the interactions between the microbiome and human host.

**FIGURE 1:**
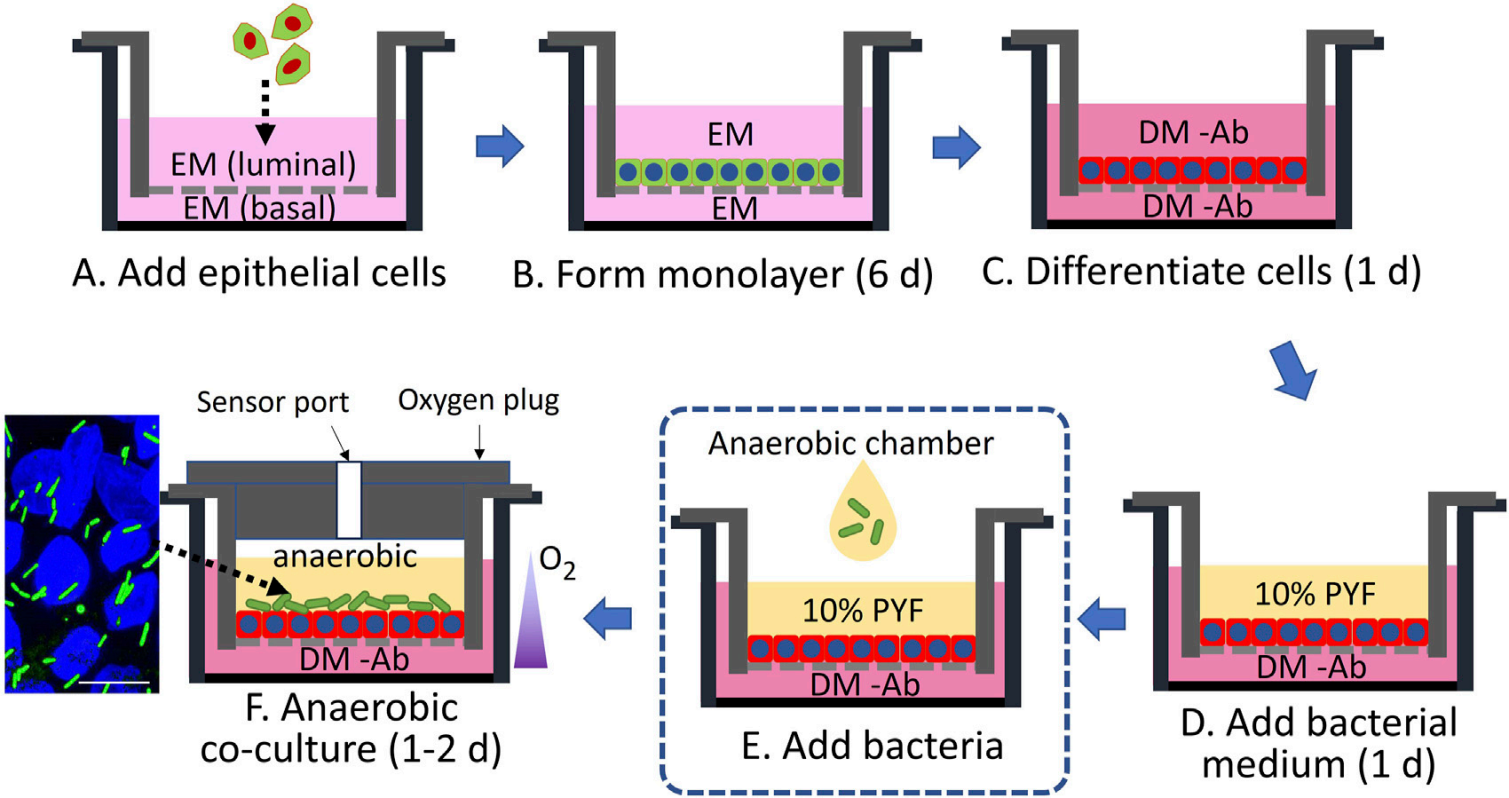
Schematic of co-culture of primary human colonic epithelial cells with bacteria. EM is a medium rich in growth factors to support stem cell expansion (green cells with blue nuclei), while DM -Ab is an antibiotic-free medium without stem-cell supportive growth factors to support the formation of differentiated cells (red cells with blue nuclei). PYF supports the growth of bacteria (green rods). The fluorescence micrograph (far lower left) shows the co-culture of differentiated colonic epithelial cells (Hoechst 33342-stained nuclei in blue) and *C. scindens* (green rods). The scale bar represents 10 µm.

*Clostridial* species such as *C. scindens* tolerate O_2_ concentrations up to 2%–3% (Morvan et al., 2021; Chia, 2023), but higher concentrations are lethal. These obligate anaerobes are able to survive in the environment of the luminal-facing differentiated colonic epithelium that exists in physiologic hypoxia of <1–3% O_2_. In contrast, the O_2_ concentration increases to 10% near the colonic epithelial cells at the crypt base, *i.e.*, deeper in the submucosa, where the stem-cell niche produces proliferative cells to replenish the epithelial layer (Keeley and Mann, 2019; Schwerdtfeger et al., 2019). To achieve these distinct O_2_ conditions, a modified cell culture cassette for O_2_-gradient formation was previously demonstrated for co-culture of epithelial cells and anaerobes (Kim et al., 2019; Kim et al., 2022). This cassette, based on a hanging basket, possessed luminal and basal reservoirs so that both sides of the colonic epithelial cell culture were accessible. To create an O_2_ gradient across the tissue surface, the side walls of the luminal compartment were impervious to O_2_ and an O_2_ impermeable plug was inserted into the luminal reservoir to prevent atmospheric O_2_ ingress. In contrast, the basal reservoir was open to the environment. Under these conditions, a confluent monolayer of epithelial cells has been shown to consume O_2_ at a sufficient rate to decrease the luminal O_2_ to <1% in 3.75 h (Kim et al., 2019). In the current experiments, epithelial stem cells were plated on the luminal side of the hanging basket’s porous membrane in the cassette and cultured in a medium rich in growth factors (expansion medium, EM) without the luminal plug so that a high O_2_ tension was maintained (Figures 1A,B). After forming a confluent monolayer, the cells were fully differentiated by removal of growth factors (differentiation medium without antibiotics, DM -Ab) producing a high-resistance epithelial monolayer (TEER = 507 ± 126 Ω·cm^2^, n = 3, Figure 1C). The luminal DM -Ab was then replaced with a minimal nutrient medium (PYF) suitable for bacterial culture (Figure 1D). The cassette was then transferred into an anaerobic chamber (Figure 1E), the plug was installed, and the assembled cassette was placed in a standard tissue-culture incubator (Figure 1F). Under these conditions, the maximal O_2_ concentration in the luminal compartment was 0.6% ± 0.4% after plug installation and decreased to a steady state value of 0.4% ± 0.1% during culture due to continued O_2_ consumption by the epithelial cells (Supplementary Figure S2). *C. scindens* was subsequently inoculated onto the intestinal epithelium at 10^3^ colony-forming unit (CFU)/ml (1,300 ± 500 cells in total). The CFU increased to 3×10^4^ ± 0.95 × 10^4^ CFU/mL (approximately 15,500 ± 5,000 in total, doubling time 13 h) after 48 h (*p* = 0.045, Supplementary Figure S3A), suggesting that *C. scindens* not only tolerated the luminal microenvironment but was able to replicate.

### 3.2 Co-culture of epithelial cells with *Clostridium scindens*

**FIGURE 2:**
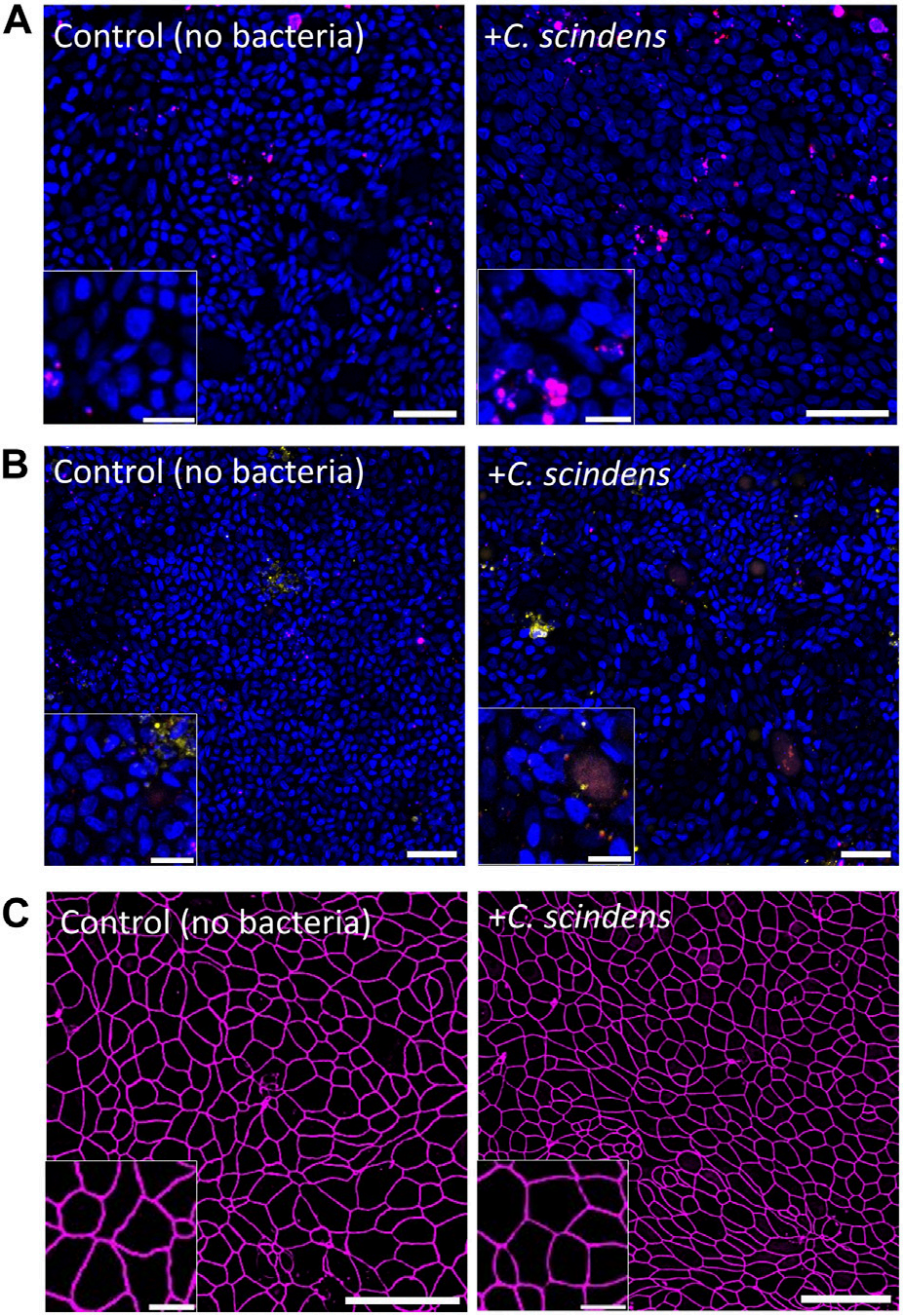
Co-culture of primary human colonic epithelial cells with *C. scindens*. **(A, B)** Fluorescence images of colonic epithelial cells stained with PI (red, panel **(A)**, annexin V (yellow, panel **(B)**, and Hoechst 33342 (blue) with and without *C. scindens* co-culture for 48 h. Scale bar = 50 µm with the inset scale bar = 10 µm. **(C)** Images of colonic epithelial cells stained for ZO-1 (magenta). Scale bar = 50 µm with the inset scale bar = 10 µm.

The *in vivo* colonic lumen acts as a host to *C. scindens* with co-existence of and mutual benefit for both the mammalian and bacterial cells. *C. scindens* ferments fiber and metabolizes bile acids to support both the health of the microbial community as well as that of the epithelial cells (Wlodarska et al., 2017; Kang et al., 2019). In turn the epithelial cells form a tight barrier to prevent the intrusion of bacteria into the tissue and collaborate with the immune system to repel invading microbes (Chen et al., 2021). To determine whether the epithelial cells might be able to co-exist with the obligate anaerobes in the absence of an immune system, *C. scindens* was cultured on a confluent, differentiated epithelial cell layer. The impact on epithelial cell viability and monolayer barrier function was then assessed. When stained with propidium iodide (PI), no significant difference in epithelial cell death was observed in the presence and absence of *C. scindens* (9.8% ± 7.5% PI + area normalized to total Hoechst 33342+ cell area for the control vs. 10.8% ± 4.2% after 48 h of co-culture with *C. scindens*, *p* = 0.64, Figure 2A; Supplementary Figure S4A). Programmed cell death was assessed using an annexin V conjugate assay to identify phosphatidylserine in the outer membrane leaflet. No significant difference in the binding of annexin V to the epithelial cells was observed in the absence and presence of *C. scindens* (9.5% ± 4.0% annexin V+ area normalized to total Hoechst 33342+ cell area for the control vs. 6.1% ± 1.6% after 48 h of co-culture with *C. scindens*, *p* = 0.24, Figure 2B; Supplementary Figure S4B). Barrier integrity was measured by assaying the TEER after 48 h of culture with and without *C. scindens*. In both instances the TEER decreased over time but was not significantly different (control 168 ± 18 vs. 150 ± 37 Ω·cm^2^ with *C. scindens*, *p* = 0.45, Supplementary Figure S5A). Since TEER represents a global measurement of barrier function, the integrity of cell-cell junctions was also assessed by fluorescent staining for ZO-1, which is a constituent of tight junctions located on the cytoplasmic membrane surface (McNeil et al., 2006). When visualized by microscopy, cells with and without *C. scindens* co-cultured for 48 h possessed similar ZO-1 staining patterns (Figure 2C). When the area, circularity, and perimeter of the cells was measured, no significant differences were identified for the epithelial cells with and without *C. scindens* (area: 77 ± 19 μm^2^ without vs. 70 ± 15 μm^2^ with, *p* = 0.3; circularity: 0.78 ± 0.02 without vs. 0.79 ± 0.02 with, *p* = 0.2; perimeter: 34 ± 5 µm without vs. 32 ± 4 µm with, *p* = 0.3, N = 3 technical replicates, Supplementary Figure S6). These data suggest that although the number of *C. scindens* expanded 10-fold, the epithelial cells did not die or lose barrier integrity during the 48 h of co-culture. However, extremely high numbers of inoculated *C. scindens* (∼5×10^6^ cells) did result in an increase in epithelial cell death at 48 h of co-culture (Supplementary Figure S7).

### 3.3 Measurement of epithelial cell inflammation in the presence of *Clostridium scindens*

IL-8 and MCP-1 are chemokines produced by epithelial cells in response to bacterial products and are often used as markers of an epithelial cell inflammatory response (Deshmane et al., 2009; La Pine and Hill, 2011). The concentrations of IL-8 and MCP-1 in the basal media were measured in the absence and presence of *C. scindens* co-culture after 48 h. The concentration of IL-8 was not significantly different in the two culture conditions (687 ± 149 pg/mL without vs. 789 ± 199 pg/mL with *C. scindens*, *p* = 0.38, Supplementary Figure S8A). Likewise, the concentration of MCP-1 was not significantly different (9.8 ± 2.1 pg/mL without vs. 7.4 ± 1.8 pg/mL with *C. scindens*, *p* = 0.21, Supplementary Figure S8B). The concentration of IL-8 secreted into the basal reservoir was similar to that in previous studies (300–600 pg/mL) (Wang et al., 2018b; Kim et al., 2022). Additionally, the concentration of MCP-1 was near that reported for colonic epithelial cell cultures (6.9–27 pg/mL) (Futagami et al., 2003; Fujita et al., 2010). Under these conditions, the co-culture of *C. scindens* did not alter the inflammatory response of the colonic cells.

### 3.4 Metabolism of cholic acid by *Clostridium scindens*

**FIGURE 3:**
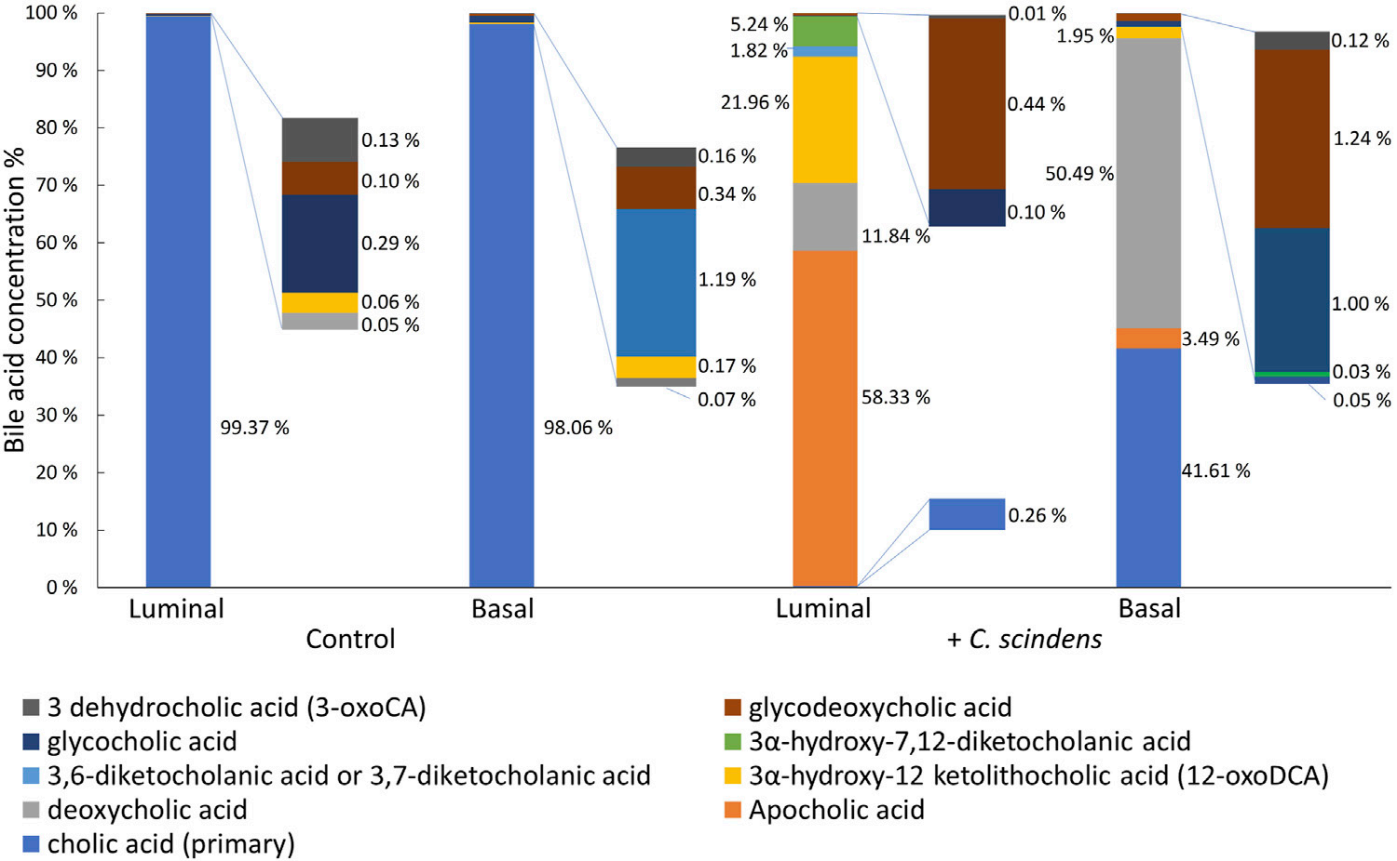
Formation of cholic acid metabolites. Cholic acid was added to the luminal compartment of the hanging basket with cultured epithelial cells in the absence and presence of *C. scindens*. The metabolites were measured in the luminal and basal compartments of the cassette by mass spectrometry.

Bile acids flow from the liver into the small intestine via the bile duct. The major portion of bile acids (∼80%) are actively transported back to liver in the terminal ileum. However, ∼15% of bile acids transit through the digestive tract to reach the colon and are then metabolized by bacteria into secondary bile acids that diffuse passively across the colonic epithelium to return to the liver (Di Ciaula et al., 2017). *C. scindens* is known to convert primary bile acids such as cholic acid into a variety of secondary bile acids (*e.g.*, deoxycholic acid and lithocholic acid) in the colon (White et al., 1980; Ridlon et al., 2014; Kang et al., 2019). To assess bile acid conversion by *C. scindens*, cholic acid (200 µM) was added in the luminal reservoir in the presence of epithelial cells with or without *C. scindens* and the concentrations of cholic acid and its metabolites were measured by mass spectrometry (Figure 3). In the absence of *C. scindens*, only cholic acid (99.37% of total luminal bile acids) was found in the luminal reservoir. In these cultures, cholic acid was also detected in the basal compartment (98.06% of total basal bile acids). In contrast, the luminal reservoir of epithelial cells co-cultured with *C. scindens* demonstrated a range of secondary bile acids (>99% conversion) with the predominant being apocholic acid (58.33% of total luminal bile acids), which is a byproduct of the dehydration of cholic acid (Shi et al., 2023). Other prominent metabolites identified in the luminal compartment included 3α-hydroxy-12 ketolithocholic acid (21.96% of total luminal bile acids) and deoxycholic acid (11.84% of total luminal bile acids). These two metabolites are formed via a dehydroxylation pathway of *C. scindens* and have been observed previously *in vitro* and *in vivo* (Marion et al., 2019; Funabashi et al., 2020). The basal compartment possessed a distinct pattern of bile acids. The predominant secondary bile acid was deoxycholic acid (50.49% of total basal bile acids) in the presence of *C. scindens*, but untransformed cholic acid was also present (41.61% of total basal bile acids) as were other products. The presence of the bile acids in the basal compartment was most likely due to passive diffusion as occurs in the colon *in vivo* (Mekhjian et al., 1979). These data suggest that molecular interactions between *C. scindens*-epithelial cells, such as bile acid metabolism were present in this *in vitro* system.

### 3.5 Co-culture of colonic epithelial cells with the intestinal pathogen *Staphylococcus aureus*

**FIGURE 4:**
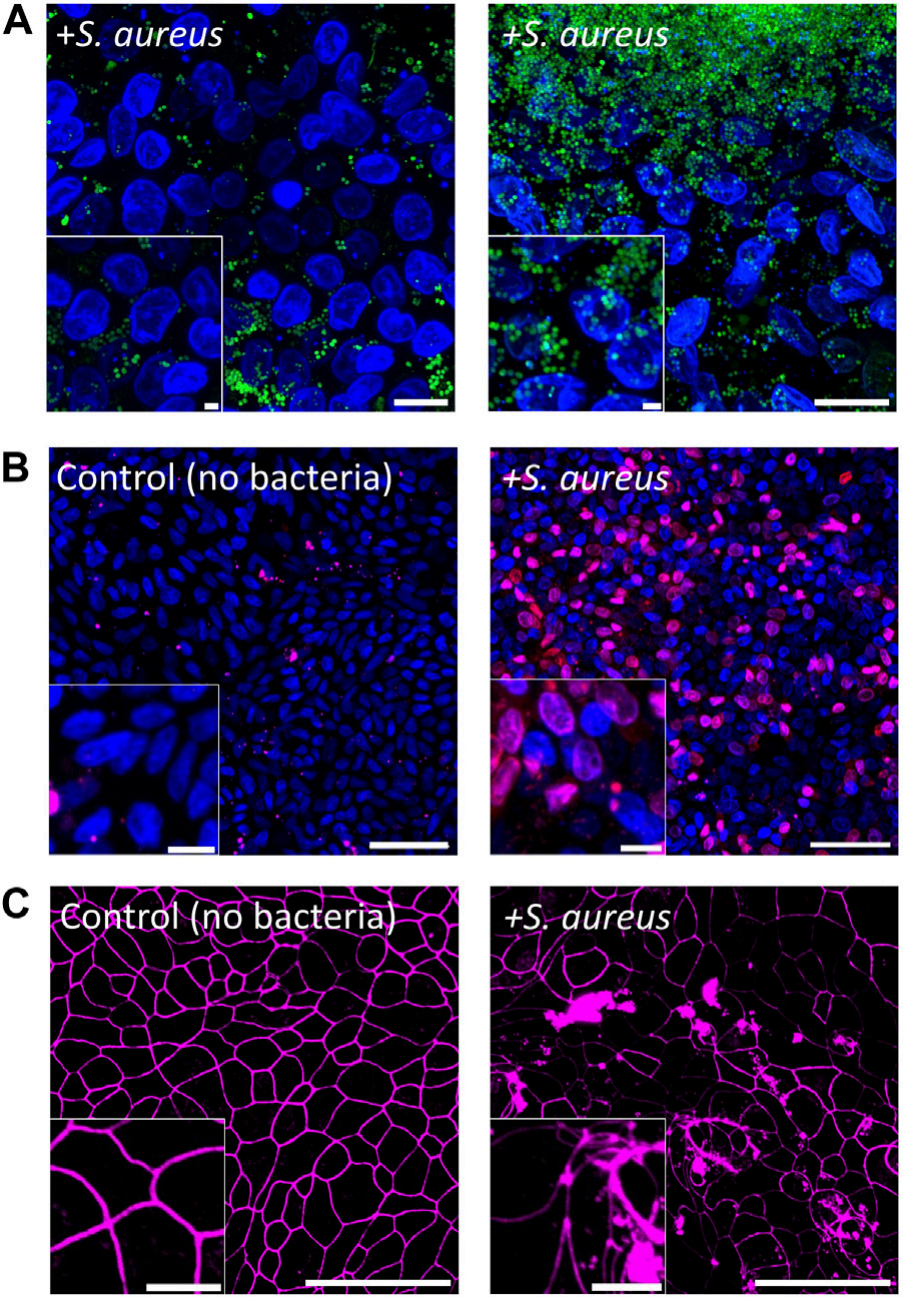
Co-culture of colonic epithelial cells with *Staphylococcus aureus*. **(A)** Fluorescence images of epithelial cell co-culture with *S. aureus* after 1 d. Colonic epithelial cell nuclei were stained with Hoechst 33342 (blue), and *S. aureus* was stained with SYTO9 (green). Scale bar = 10 µm and the inset scale bar is 2 µm. **(B)** Images of colonic epithelial cells stained with PI (red) and Hoechst 33342 (blue) cultured with and without *S. aureus* for 1 d. Scale bar = 50 µm and the inset scale bar = 10 µm. **(C)** Images of colonic epithelial cells stained with ZO-1 (magenta) when cultured with and without *S. aureus*. Scale bar = 50 µm and the inset scale bar = 10 µm.

*S. aureus* is a facultative anaerobe normally found on human skin and within the nasal cavities but can be an opportunistic pathogen of the colon (O'Gara, 2017). *S. aureus* is able to produce a range of toxins including enterotoxins (SEs), hemolysins, and leukotoxins (Kong et al., 2016) so that colonization of the colon by *S. aureus* can lead to severe nausea, vomiting, and diarrhea (CDC, 2018). To examine the cytotoxicity of *S. aureus* to colonic epithelial cells, we inoculated *S. aureus* onto the epithelial monolayer at 900 ± 370 CFU/mL (450 ± 190 cells in total). After 24 h of co-culture in the anaerobic cassette, *S. aureus* numbers increased to 1.4×10^8^ ± 6 × 10^7^ CFU/mL (7.0×10^7^ ± 2.8×10^7^ total cells), which was significantly higher than the initial inoculum (*p* = 0.02, Figure 4A; Supplementary Figure S3B) and represents a doubling time of ∼83 min. Growth of *S. aureus* on the epithelial cell culture was substantially more robust than that of *C. scindens* (Moretó and Pérez-Bosque, 2009; Martens et al., 2021). As the epithelial cells died as a result of co-culture with *S. aureus*, many nutrients may have become available to benefit the growth of *S. aureus*. In these co-cultures, *S. aureus* grew both in a dispersed manner as well as in clusters or sheets of bacteria fully covering the monolayer, suggesting the formation of biofilms.

**FIGURE 5:**
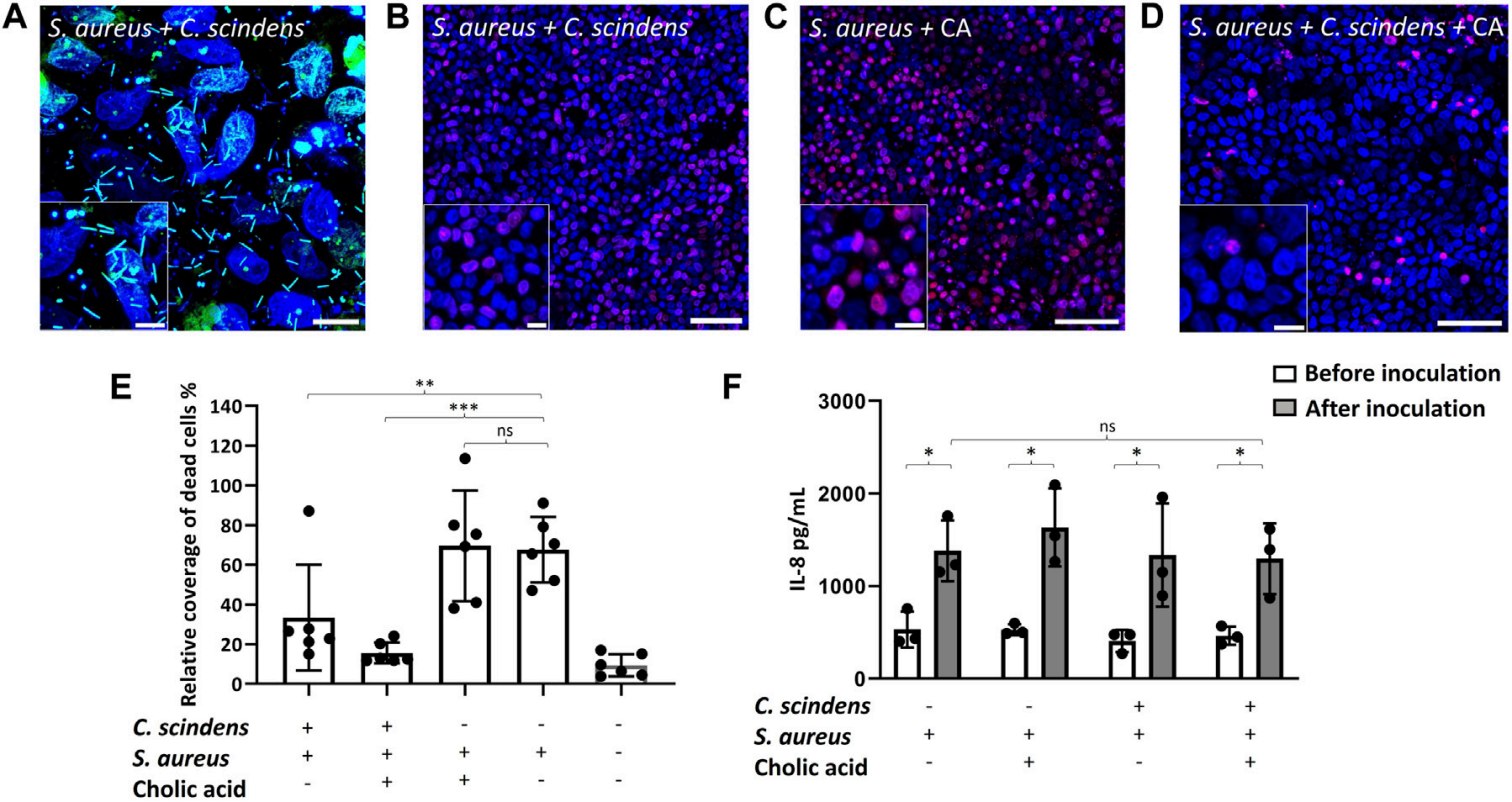
Co-culture of colonic epithelial cells with *C. scindens* and *S. aureus*. **(A)** Images of the co-culture. The nuclei of colonic epithelial cells were stained with Hoechst 33342 (blue), and *S. aureus* and *C. scindens* were stained with SYTO9 (green). Scale bar = 10 µm and the inset scale bar = 5 µm. **(B–D)** Images of colonic epithelial cells stained with PI (red) and Hoechst 33342 (blue) co-cultured with *S. aureus* + *C. scindens* **(B)**, *S. aureus* + 200 µM cholic acid **(C)**, and *S. aureus* + *C. scindens* + 200 µM cholic acid **(D)**, scale bar = 50 µm and the inset scale bar = 10 µm. **(E)** Measurement of dead cells in the absence and presence of *C. scindens*, *S. aureus*, and/or cholic acid. The Y-axis is the PI-positive area normalized to the Hoechst 33342-positive area, N = 6 technical replicates. **(F)** The IL-8 concentration was measured in the basal media before (open bars) and after (closed bars) co-culturing with *S. aureus*, *C. scindens*, and/or cholic acid (200 mM). N = 3 technical replicates.

The impact of *S. aureus* on the epithelial cells was assessed by measuring epithelial cell death and monolayer barrier integrity. Compared to control cultures, the viability of colonic epithelial cells in the presence of *S. aureus* was significantly decreased after 24 h of co-culture (9.8% ± 7.5% PI + area normalized to total Hoechst 33342+ cell area for the control vs. 68% ± 16% after co-culture with *S. aureus*, *p* = 0.0007, Figure 5E). Visually, a majority of the epithelial cells appeared dead in the cultures (Figure 4B). This is consistent with the measured TEER of 70 ± 12 Ω·cm^2^ (*p* < 0.001 relative to control, Supplementary Figure S5B), indicating a complete loss of barrier function in the presence of *S. aureus* after 24 h. To examine cell-cell tight junctions, the epithelial monolayers were stained for ZO-1, which showed a loss of cell boundaries and an appearance of large aggregates of ZO-1 in the colonic cells. This finding indicated that the intercellular tight junctions were disrupted in these cultures (Figure 4C). Further confirmation was obtained by quantification of the epithelial cells’ morphology with the area, perimeter, and circularity all significantly different compared to that of colonic cells cultured in the absence of *S. aureus* (area: 77 ± 19 μm^2^ without vs. 161 ± 43 μm^2^ with, *p* < 0.001; circularity: 0.78 ± 0.02 without vs. 0.69 ± 0.04 with, *p* < 0.001; perimeter: 34 ± 5 µm without vs. 54 ± 7 µm with, *p* < 0.001, N = 3 technical replicates, Supplementary Figure S6). The area and perimeter of the epithelial cells increased in the presence of *S. aureus* while the circularity decreased, in agreement with the observed distorted cell boundaries on the micrographs. Consistent with these results, the IL-8 concentration in the basal compartment of the epithelial cells was significantly increased after 24 h of *S. aureus* co-culture compared to that for the epithelial cells alone (531 ± 196 pg/mL vs. 1,382 ± 329 pg/mL, *p* = 0.02, Figure 5F). It is noteworthy that *S. aureus* was inoculated onto the epithelial monolayer at a 2-to-3-fold lower CFU than *C. scindens*. The shorter *S. aureus* doubling time and potentially toxin production under these conditions enabled *S. aureus* to expand in number rapidly, leading to a cellular inflammatory response, cell death, and destruction of the monolayer barrier integrity.

### 3.6 Co-culture of epithelial cells with *Clostridium scindens* and *Staphylococcus aureus*

As a commensal strain, *C. scindens* exerts a protective effect on the colon by converting 7α-dehydroxylating primary bile acids to form secondary bile acids which then inhibit pathogenic bacterial growth (Greathouse et al., 2015; Kang et al., 2019). To explore this defensive behavior of *C. scindens*, epithelial monolayers were colonized with *C. scindens* by co-culture for 24 h. *S. aureus* was then inoculated onto the *C. scindens*/epithelial cell co-culture (Figure 5A). After an additional 24 h of incubation, epithelial cell death, cell morphology, and secreted cytokines were measured. Pre-culture of the epithelial cells with *C. scindens* significantly enhanced the viability of the epithelial cells after addition of *S. aureus* (*p* = 0.02, Figures 5B,E). Remarkably, epithelial cell death in the presence of both *C. scindens* and *S. aureus* was not significantly different from epithelial cells in the absence of bacteria (*p* = 0.18, Figure 5E). Furthermore when ZO-1 immunofluorescence was measured, the presence of pre-cultured *C. scindens* also reduced the alterations in cell morphology in the co-culture with *S. aureus* compared to culture in the absence of *C. scindens* (area: 161 ± 43 μm^2^ with *S. aureus* only vs. 116 ± 30 μm^2^ with both, *p* = 0.01; circularity: 0.69 ± 0.04 with *S. aureus* only vs. 0.74 ± 0.03 with both, *p* = 0.003; perimeter: 54 ± 7 µm with *S. aureus* only vs. 43 ± 7 µm with both, *p* = 0.008, N = 3 technical replicates, Supplementary Figures S6, S10). The presence of *C. scindens*, however, did not significantly alter the amount of IL-8 secreted in the presence of *S. aureus* alone (*p* = 0.8, Figure 5F) suggesting that the epithelium was still inflamed by the presence of the *S. aureus*, but protected from death by the co-culture with *C. scindens*. The measured *S. aureus* CFU in the absence or presence of *C. scindens* was not significantly different (7.0×10^7^ ± 2.8×10^7^ without vs. 6.3×10^7^ ± 2.4×10^7^ with *C. scindens*, *p* = 0.83, Supplementary Figure S3B), therefore repression of *S. aureus* growth was not an explanation for the increased epithelial cell survival, which was affirmed when the two bacteria were co-cultured in the absence of an epithelium (Kang et al., 2019).

Secondary bile acids are reported to diminish the negative impact of *S. aureus* by disruption of the bacterium’s membrane (Sannasiddappa et al., 2017), and by minimizing excessive inflammation through inhibition of NF-κB and NLRP3 pathways (Zhao et al., 2023). Cholic acid (200 µM) was added to the luminal compartment during *C. scindens* colonization to enable the formation of secondary bile metabolites. *S. aureus* was then added, and epithelial cell death and IL-8 secretion were measured after 24 h. Epithelial cell death was not significantly decreased by the addition of cholic acid to the *C. scindens-S. aureus* mixed culture (*p* = 0.13, Figure 5B,D,E). IL-8 production was also not significantly altered in the presence of cholic acid relative to that without cholic acid (*p* = 0.92, Figure 5F). Cholic acid added to epithelial monolayers with only *S. aureus* (no *C. scindens*) did not significantly alter epithelial cell viability (*p* = 0.88) or IL-8 production (*p* = 0.46) compared to *S. aureus* only co-cultures (Figure 5C,E,F). The measured *S. aureus* CFU in the absence or presence of *C. scindens* + cholic acid was not significantly different (7.0×10^7^ ± 2.8×10^7^ without vs. 6.6×10^7^ ± 3.8×10^7^ with *C. scindens*/cholic acid, *p* = 0.93, Supplementary Figure S3B), so repression of *S. aureus* growth was again not a likely explanation for the improved epithelial cell survival. These results depart from the findings of prior studies in which bile acids repressed the growth of *S. aureus* (Sannasiddappa et al., 2017; Kang et al., 2019); however, we note that epithelial cells were absent from those experiments. For this reason, we cultured *S. aureus* in the presence of cholic acid and deoxycholic acid but without epithelial cells. We observed a significantly decreased growth rate as measured by O.D. (Supplementary Figure S9). It is likely that the epithelial cells, *C. scindens*, or their metabolic products other than bile acids are acting in a complex manner to impact *S. aureus* growth.

### 3.7 Measurement of *Staphylococcus aureus* toxin in the presence of *Clostridium scindens* and epithelial cells

**FIGURE 6:**
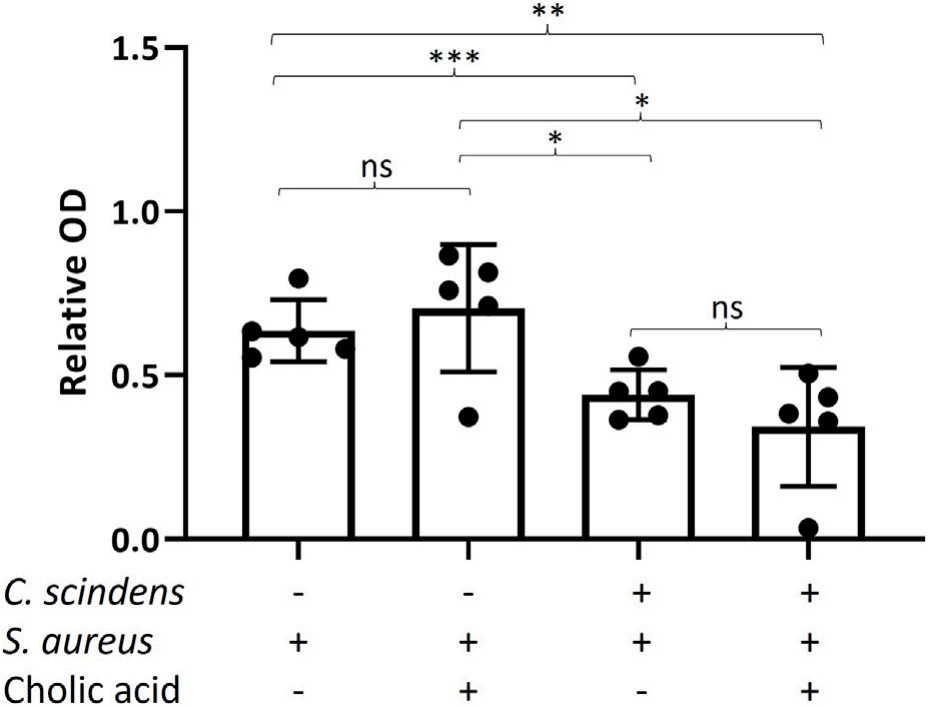
Measurement of total enterotoxin produced by *S. aureus* in the absence and presence of *C. scindens* ± cholic acid. The Y-axis is the relative optical density (O.D., 420 nm) normalized to an enterotoxin standard, N = 5 technical replicates. *: *p* < 0.05, **: *p* < 0.01, ***: *p* < 0.001.

*S. aureus* enterotoxins (SEs) are potent exotoxins that damage colonic epithelial cells by stimulating the secretion of cytokines and provoking inflammation (Aljasir and D’Amico, 2020; Cretenet et al., 2011). Enterotoxin B (SEB) also disrupts the integrity of epithelial barriers, initiating cell death (Martens et al., 2021). To determine whether *C. scindens* reduces the cytotoxicity of *S. aureus* by altering toxin formation, enterotoxin (SE) production by *S. aureus* was measured in the luminal reservoir. For simplicity, the selected assay measured total enterotoxin (SEA, SEB, SEC, and SED). Addition of *C. scindens* with or without cholic acid significantly diminished total SE production by *S. aureus* compared to cultures without *C. scindens* pre-culture (*p* = 0.007, *C. scindens*; *p* = 0.012 *C. scindens* + cholic acid) (Figure 6). Addition of cholic acid to the luminal reservoir of *S. aureus*/epithelial cell cultures in the absence of *C. scindens* did not significantly alter toxin production compared to the cultures without cholic acid (*p* = 0.5) suggesting that cholic acid itself did not modulate toxin production (Figure 6). These results indicate that the production of SE by *S. aureus* was suppressed when *C. scindens* was present, but this suppression was not reliant on the creation of secondary bile acids. Similar findings have been observed in co-cultures of *S. aureus* with other bacterial species *in vitro* (Zdenkova et al., 2016; Nogueira Viçosa et al., 2019; Rodríguez-Sánchez et al., 2022). However, it is noteworthy that this phenomenon was observed for the first time in *S. aureus-C. scindens* co-culture in the presence of colonic cells.

## 4 Discussion

Culture innovations were made to maintain the viability of epithelial cells in culture with bacteria for 48 h when using the O_2_ gradient cassette. In contrast, prior cultures of the epithelial cells in the O_2_ gradient cassette were heavily damaged at 24 h (4, 7). Additionally, prior reports used only a single, probiotic, nonpathogenic bacteria due to the very short lifespan of the epithelial cells under the conditions used. In this study, the obligate anaerobic commensal *C. scindens* was co-cultured with primary human colonic epithelial cells under an O_2_ gradient. The O_2_ gradient enabled survival of the obligate anaerobic bacteria in the anaerobic microenvironment while providing adequate O_2_ concentration for the support of the human cells. Compatibility between the two species (human and microbe) over a 48-h period was shown by the high cell viability, excellent barrier function, appropriate cell morphology, and continued cytokine production. The extended epithelial cell lifetime enabled pre-colonization of the epithelium with a probiotic (*C. scindens*) followed by introduction of a pathogen strain (*S. aureus*) to mimic the sequence of events occurring during intestinal infection *in vivo*. The O_2_ gradient cassette is a simple, inexpensive, easy-to-use device that does not require a flow system so that this culture system with extended lifetime is accessible to nonexperts.

When the intestinal pathogen *S. aureus* was inoculated in the luminal compartment at lower density than *C. scindens* under comparable conditions, greater than 60% of the epithelial cells died within 24 h. When *S. aureus* was inoculated after establishing an epithelial cells-*C. scindens* co-culture, death of the epithelial cells was greatly reduced and was not significantly different compared with the amount of cell death in the absence of bacteria. These results suggest that the toxicity of *S. aureus* was reduced in the presence of *C. scindens*. Although the growth of *S. aureus* was not noticeably repressed in the presence of *C. scindens*, the concentration of enterotoxin decreased by nearly 50%. *C. scindens* has been reported in multiple *in vitro* studies and an animal study to have an antimicrobial effect on intestinal pathogenic strains (Buffie et al., 2015; Greathouse et al., 2015; Kang et al., 2019; Zhao et al., 2023). The co-culture results here demonstrate a similar outcome in the presence of primary human colon cells within our *in vitro* platform. These data suggest that the platform could be used to expand the study of antimicrobial effects of intestinal probiotics in an environment more closely mimicking the *in vivo* colonic environment.

Bile acids are known to play important roles in modulating the intestinal microbiome. *C. scindens* converts primary bile acids (cholic and chenodeoxycholic acid) to secondary bile acids (*e.g.*, deoxycholic acid and lithocholic acid) in the intestine (Greathouse et al., 2015; Kang et al., 2019). Furthermore, secondary bile acids affect lipid membranes similarly to detergent (Kumar et al., 2020) and are believed to serve an antimicrobial role within the intestinal microbiome (Ridlon et al., 2016). In the current study, although *C. scindens* was shown to convert cholic acid to secondary bile acids in the luminal medium, the growth of *S. aureus* was not significantly affected by these bile acid products. This finding was in contrast to prior antimicrobial studies using bile acid supplemented medium, which demonstrated reduced *S. aureus* growth (Supplementary Figure S9). Of note is that these studies did not utilize *C. scindens* for bile acid conversion and epithelial cells were not present. One explanation for the different outcomes could be the transport of secondary bile acids from the luminal to the basal compartment across the epithelial monolayer. In the intestine, >95% of bile acids are re-absorbed and then delivered to the liver through the portal vein (Ticho et al., 2019). In the current studies, the majority of deoxycholic acid, which has a stronger antimicrobial effect on *S. aureus* compared to other secondary bile acids (Kang et al., 2019), was detected in the basal media. Thus, the decreased concentration of bile acid in the luminal reservoir may have reduced its antimicrobial impact on *S. aureus.* In the presence of the cholic acid in the cultures the dominant species in the basal medium was deoxycholic acid while the apocholic acid was the majority metabolite in the luminal compartment. Apocholic acid is an uncommon bile acid found in human feces and known to be a carcinogenic byproduct resulting from the mild dehydration of cholic acid facilitated by bacterial dehydrogenases (Shi et al., 2023). While there are no reports of apocholic acid production by *C. scindens*, our findings suggest that the deoxygenation pathway of intestinal bacteria may be influenced by the presence of host cells, a phenomenon not observed in single strain cultures.

Enterotoxin is known to trigger an immune response that leads to damage of intestinal mucosal cells and diarrhea (Fayyaz et al., 2022). At least 20 types of *S. aureus* enterotoxins with a molecular size of ∼25 kDa (220–240 amino acids) have been identified (Pinchuk et al., 2010). The production of enterotoxins is dependent on a variety of parameters, including temperature, pH, and the presence of other microorganisms (Cretenet et al., 2011; Perales-Adán et al., 2018). Recent studies have shown that probiotic isolates can alter biochemical conditions to repress *S. aureus* enterotoxin production in bacterial co-culture *in vitro* without significantly inhibiting the growth of *S. aureus* (Aljasir and D’Amico, 2020). Similar results were observed here in the presence of colonic epithelial cells. Further studies on the role of probiotics in altering the luminal environment in the presence of human epithelium and pathogenic bacteria will be valuable in gaining a better understanding of the interplay of how these cells impact enterotoxin production and activity.

In the colon, stem cells at the base of the intestinal crypts serve to replenish the epithelium as the differentiated cells undergo apoptosis within a few days of migrating to the luminal surface (Wang et al., 2018a). This process maintains a self-regenerating epithelial monolayer believed to be influenced by intestinal microbiota (Donaldson et al., 2016). The primary colonic epithelial monolayer used in this study was composed of fully differentiated cells. In the absence of co-cultured bacteria, this monolayer maintains a high TEER barrier for only 2–3 days once fully differentiated due to the natural lifetime of differentiated colonic epithelial cells and a lack of proliferative cells that would otherwise maintain the monolayer (Wang et al., 2017; Wang et al., 2018a). After this time the TEER drops as the cells begin to die without replacement. The change in TEER over time is consistent with previous studies using soft scaffolds (Gunasekara et al., 2018; Kim et al., 2019; Speer et al., 2019). Future studies are planned with long-lived (>30 days) 2D and 3D crypt models that maintain a stem-cell niche to continually support the epithelium (Wang et al., 2018a; Wang et al., 2022). These models are expected to sustain epithelial integrity for significantly longer duration and enable the study of more complex microbiota and epithelial interactions.

In this study, fully differentiated, primary human colonic epithelial cells were inoculated with either intestinal probiotic (*C. scindens*), pathogenic (*S. aureus*), or both bacterial species and co-cultured under an O_2_ gradient. The O_2_ gradient produced the necessary anaerobic environment for the obligate and facultative anaerobic bacteria while maintaining the viability and function of the mammalian cells. The epithelial monolayer retained viability and barrier integrity in the presence of *C. scindens* for 48 h but was degraded within 24 h in the presence of a smaller inoculum of *S. aureus*. Pre-culturing the epithelium with *C. scindens* reduced the concentration of enterotoxin in subsequent co-culture with *S. aureus* and improved the survival of the epithelial cells but did not inhibit the growth of *S. aureus*. The O_2_-gradient cassette provided an effective and economical platform to study the interactions of intestinal microbiota with primary human cells in an environment more closely mimicking the colonic environment and provides an easy-to-use system for studies of the gut microbiome.

## Data Availability

The raw data supporting the conclusion of this article will be made available by the authors, without undue reservation.
