# Supplementary material for: In vitro co-culture of Clostridium scindens with primary human colonic epithelium protects the epithelium against Staphylococcus aureus
Source: Front Bioeng Biotechnol. 2024 Apr 12;12:1382389. doi: 10.3389/fbioe.2024.1382389 (PMC11045926; doi:10.3389/fbioe.2024.1382389)
Supplement: Supplementary file 1 [file DataSheet1.docx]

Supplementary Material

1. **Supplementary Figures and Tables**
   1. **Supplementary Tables**

**Supplementary Table S1.** Colonic media compositions used in this study.

|  | SM | EM | DM -Ab |
| --- | --- | --- | --- |
| Advanced DMEM/F12  (Thermo Fisher) | 50% (v/v) | 50% (v/v) | 100% (v/v) |
| L-WRN conditioned medium | 50% (v/v) | 50% (v/v) | - |
| GlutaMax  (Thermo Fisher) | 1× | 1× | 1× |
| HEPES | 10 mM | 10 mM | 10 mM |
| Human EGF or mouse EGF (for Medium 2 only) (Peptrotech) | 50 ng/mL | 50 ng/mL | 50 ng/mL |
| B27 | 1× | 1× | - |
| N-acetyl cysteine  (MP bio) | 1.25 mM | 1.25 mM | - |
| Gastrin  (Anaspec) | 10 nM | 10 nM | - |
| Y-27632  (ApexBio) | 10 μM | 10 μM | - |
| A83-01  (Sigma Aldrich) | 500 nM | - | 500 nM |
| Prostaglandin E2 (PGE2)  (Cayman chemicals) | - | 10 nM | - |
| Nicotinamide  (Sigma Aldrich) | - | 10 mM | - |
| SB202190  (Selleckchem) | 3 μM | 3 μM | - |
| Primocin  (InvivoGen) | 50 μg/mL | 50 μg/mL | - |

**Supplementary Table S2.** Peptone-yeast-fructose media compositions used in this study.

|  | for 1 L | |
| --- | --- | --- |
| peptone | 20 | g |
| yeast extract | 10 | g |
| glucose | 10 | g |
| resazurin | 1 | mg |
| salt solution* | 80 | mL |
| distilled water | 910 | mL |
|  |  |  |
| sodium acetate | 5 | g |
|  |  |  |
| Hemin solution** | 10 | mL |
| Vitamin K1 solution*** | 0.2 | mL |
| L-cysteine HCl×H_2_O | 0.5 | g |
|  |  |  |
| Agar (for CFU counting) | 15 | g |

***Salt solution**

|  | for 1 L | |
| --- | --- | --- |
| CaCl_2_×2H_2_O | 0.125 | g |
| MgSO_4_×7H_2_O | 0.25 | g |
| K_2_HPO_4_ | 0.5 | g |
| KH_2_PO_4_ | 0.5 | g |
| NaHCO_3_ | 5 | g |
| NaCl | 1 | g |
| distilled water | 1000 | mL |

****Hemin solution**

Dissolve 50 mg hemin in 1 ml 1 N NaOH; make up to 100 ml with distilled water. Store refrigerated.

*****Vitamin K1 solution**

Dissolve 0.1 ml of vitamin K1 in 20 ml 95% ethanol and filter sterilize. Store refrigerated in a brown bottle.

- 1. **Supplementary Figures**

**
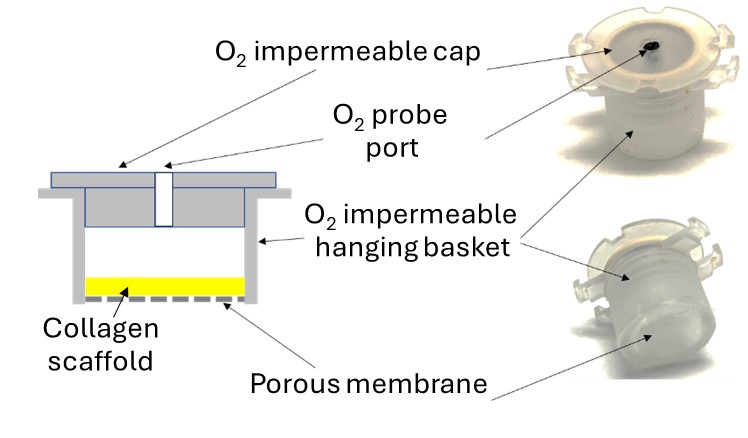
**

**Supplementary Figure S1**. Schematic (left) and images (right) of hanging basket and cap used to create an anaerobic luminal compartment. The insert was equipped with a gas-impermeable threaded plug or cap to separate the luminal compartment from the external atmosphere. A port in the plug served for measuring the oxygen saturation. The hanging basket and cap were placed into a microwell so that the basket was suspended above the base of the microwell. The unoccupied portion of the microwell was filled with medium to form a basal reservoir.


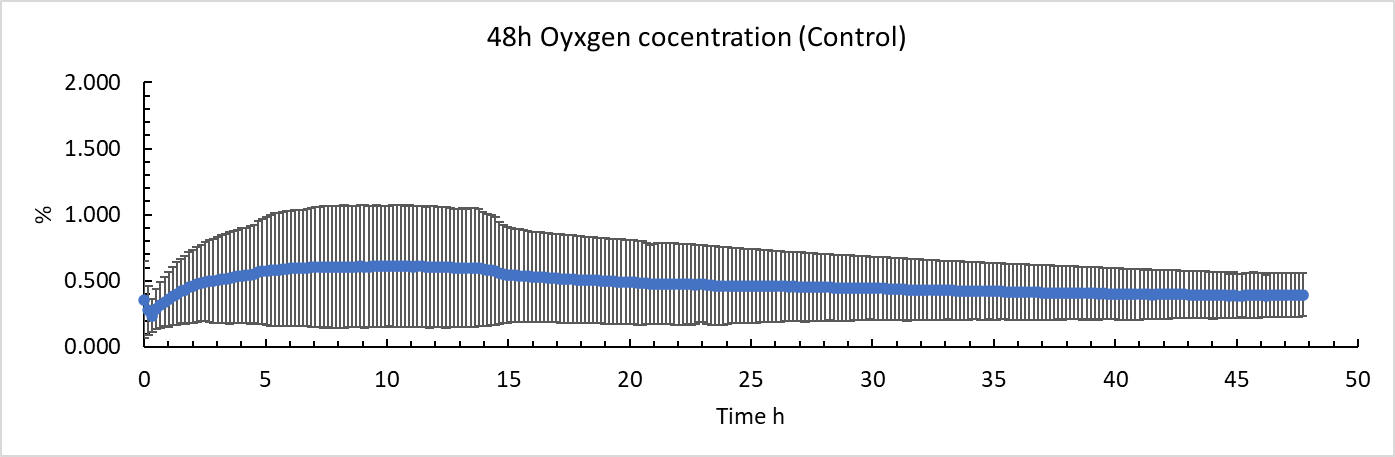


**Supplementary Figure S2.** Measured O_2_ saturation in the deoxygenated luminal media over time. Time zero was when the plug was installed. The data points represent the average of the measurements, and the error bars the standard deviation of the data (N=3 independent cultures). A port in the plug was used for insertion of an O_2_ probe, and this port was sealed with ethylene propylene diene monomer (EPDM) rubber caps (McMaster-Carr, Cat #6448K117) when an O_2_ sensor was not in place. The O_2_ probe in the luminal compartment was a needle oxygen probe (PreSens, NTH-PSt7/Microx4, Germany). The tip of the O_2_ probe was submerged into the luminal media to approximately 2 mm above the collagen surface when O_2_ was measured. The percentage of O_2_ in luminal media was determined every 10 min.

**
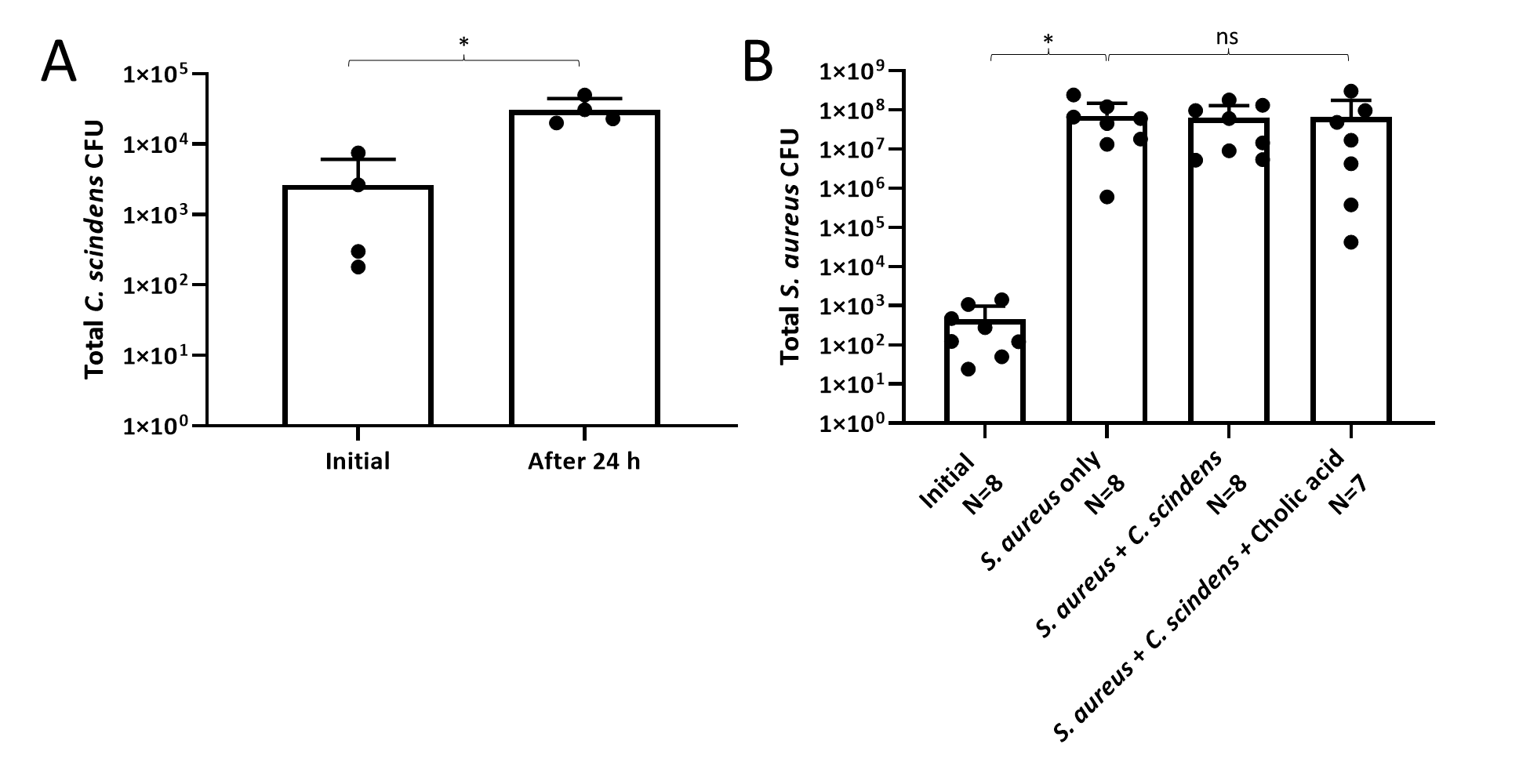
**

**Supplementary Figure S3**. The total CFU of bacterial cells in the luminal compartment before and after coculture with colonic epithelial cells. (A) The total CFU of *C. scindens* in the initial inoculation and after 48 h of coculture, N=4 technical replicates. (B) The total CFU of *S. aureus* in the initial inoculation and after 24 h of coculture. “N” provides the number of technical replicates.

**
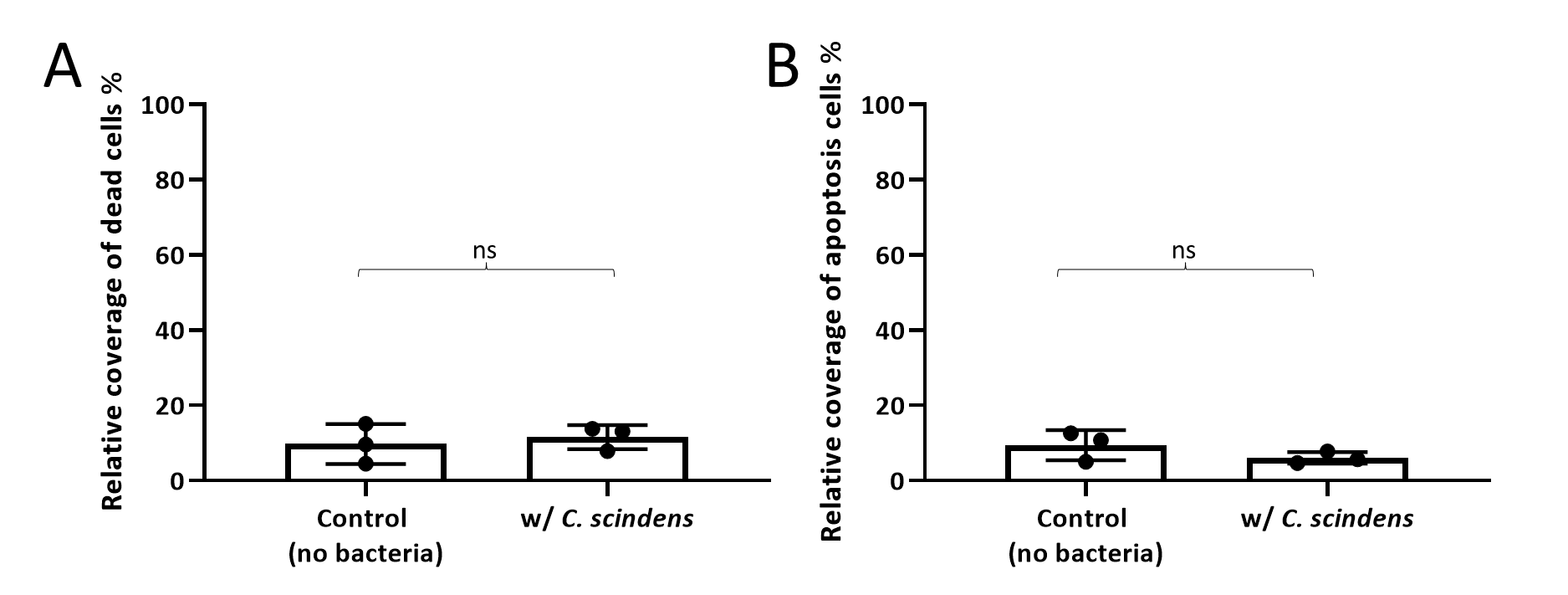
**

**Supplementary Figure S4**. Measurement of dead and apoptotic epithelial cells in the presence and absence of *C. scindens*. The Y axis is the PI-positive (A) or annexin V-positive (B) area divided by the Hoechst 33342-positive area, N=3 technical replicates.

**
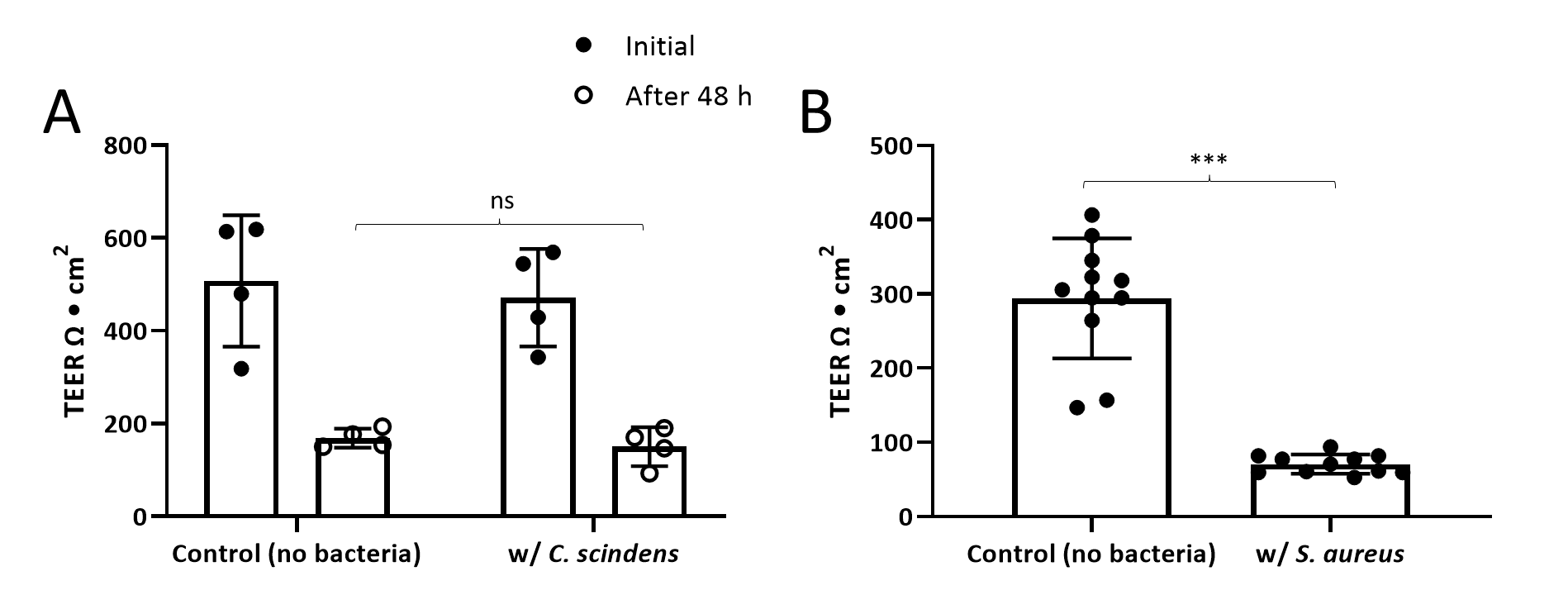
**

**Supplementary Figure S5**. TEER of epithelial layer in the cocultures with bacteria. (A) TEER of the epithelial cell layer at zero (initial) and after 48 h of coculture with or without *C. scindens*, N=4 technical replicates. (B) TEER of the epithelial cell layer at zero (initial) and after 48 h of coculture with or without *S. aureus*, N=11 technical replicates.

**
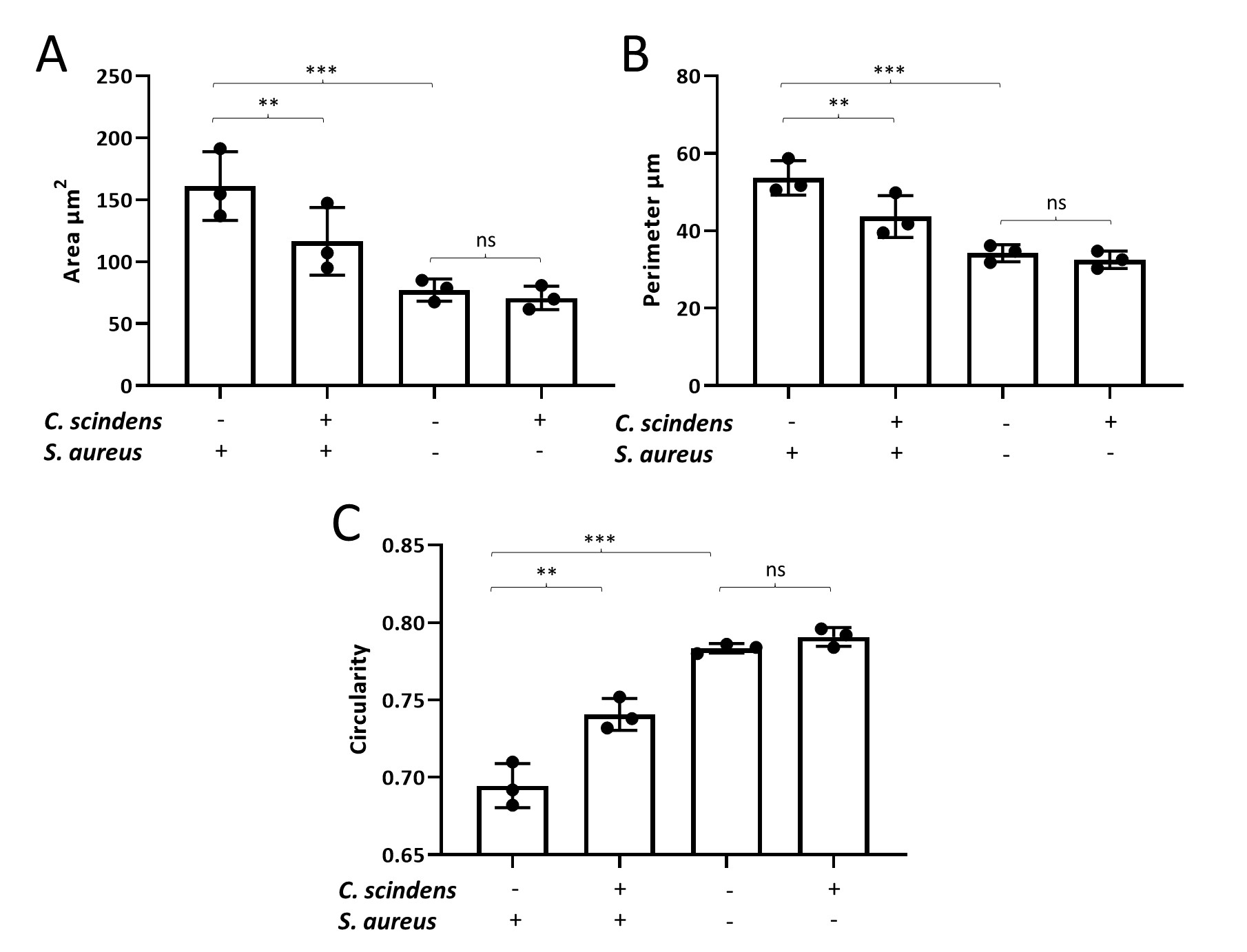
**

**Supplementary Figure S6**. Comparisons of cellular area (A), perimeter (B), and circularity (C) of colonic epithelial cells in absence and presence of *C. scindens*, *S. aureus* or both bacteria, N=3 technical replicates.


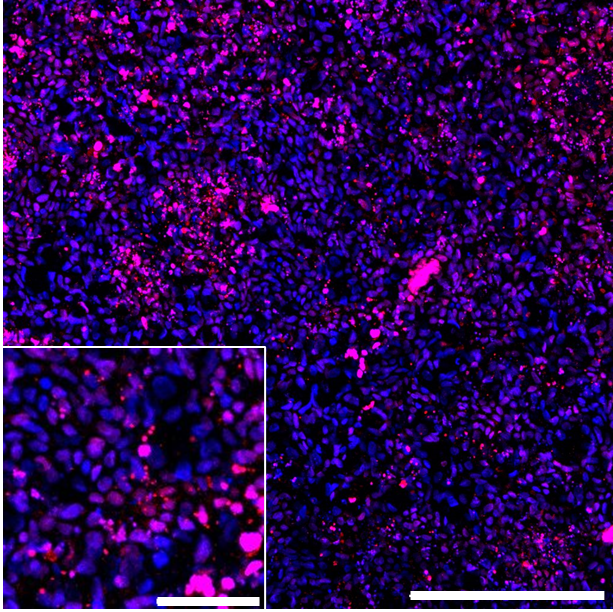


**Supplementary Figure S7**. 24 h co-culture of primary human colonic epithelial cells stained with PI (red) and Hoechst 33342 (blue) cocultured with a high initial population (5×10^6^ cells) of *C. scindens*. The *C. scindens* cells from a 10 mL culture (48 h) were washed and resuspended in 1 mL 10% PYF in PBS medium (10× concentration). The concentrated *C. scindens* suspension was then transferred to the luminal compartment of the hanging basket. *C. scindens* was then co-cultured with the colonic epithelial cells in the O2 gradient cassette. After 48 h of co-culture, the ratio of PI+ area to Hoechst 33342+ area was 77 ± 10% suggesting a higher death rate for the epithelial cells under these conditions.

**
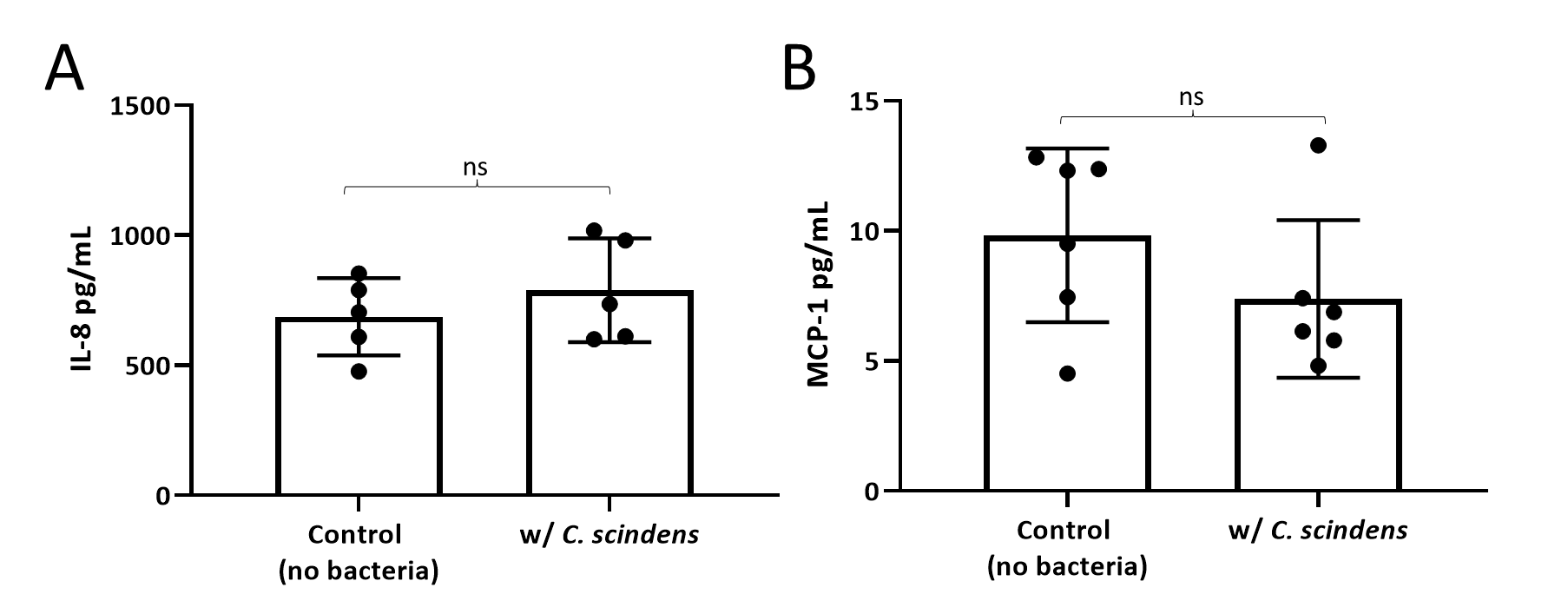
**

**Supplementary Figure S8**. IL-8 (A) and MCP-1 (B) concentrations were measured in the basal media with and without *C. scindens* coculture after 48 h. N=5 (A) and 6 (B) technical replicates.


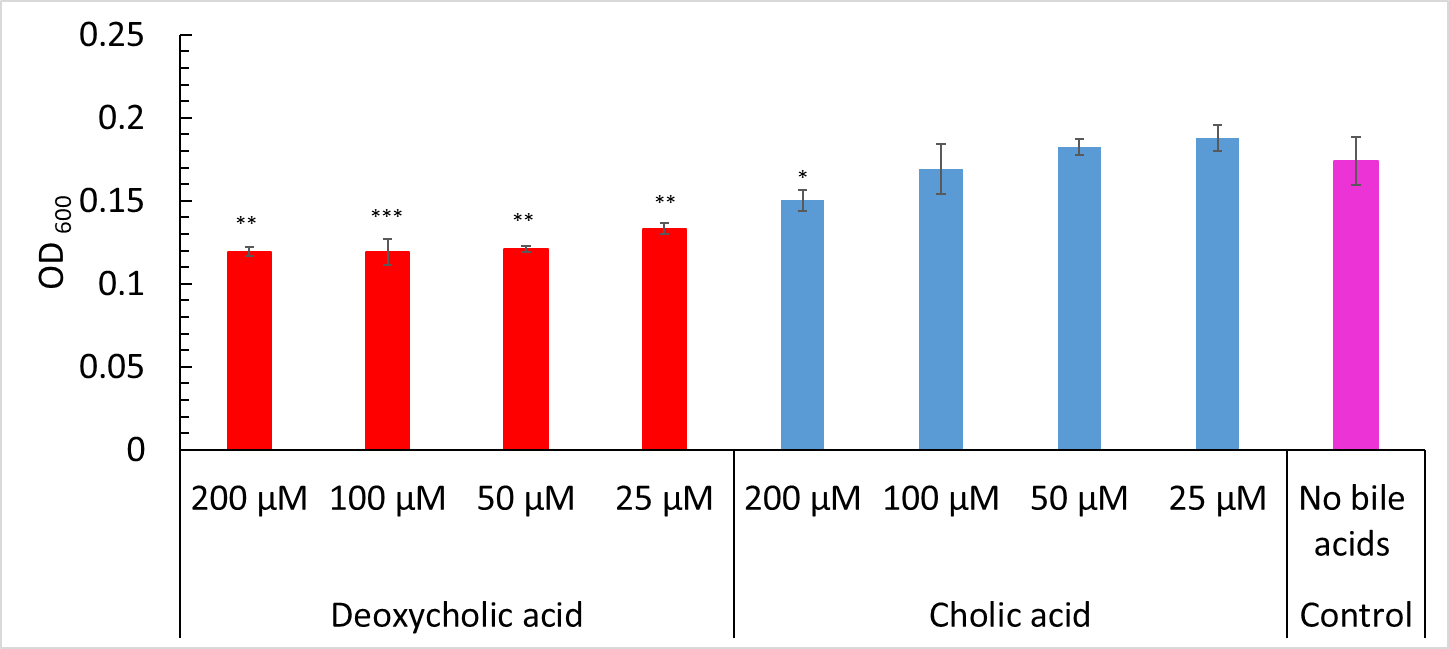


**Supplementary Figure S9**. The optical densities of 24 h *S. aureus* culture in the presence of deoxycholic acid, cholic acid or control (N=4 independent cultures). The *S. aureus* cells were washed and diluted with deoxygenated 10% PYF in PBS medium to the same initial concentration as the co-culture experiments. Next, the *S. aureus* culture was transferred to a 96-well plate with 200 µL in each well. The deoxycholic or cholic acid was added to the culture at a final concentration of 25, 50, 100 or 200 µM. The *S. aureus* cultures were then incubated anaerobically for 24 h followed by optical density measurement with a plate reader. The control samples were *S. aureus* culture without bile acids.

**
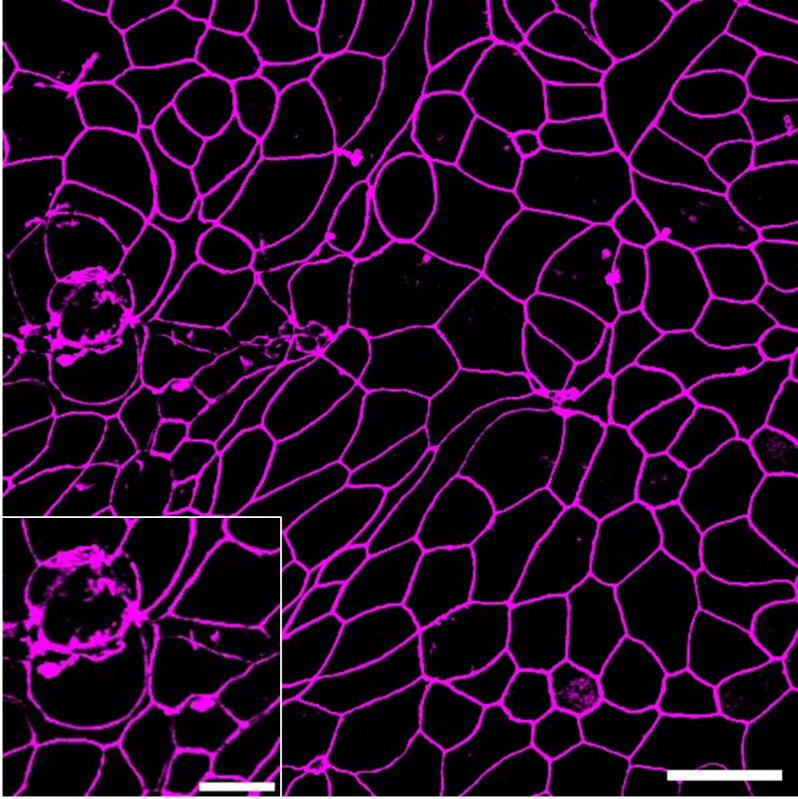
**

**Supplementary Figure S10**. Microscopy image of colonic epithelial cells stained by immunofluorescence for ZO-1 (magenta) when cultured with both *C. scindens* and *S. aureus*. Scale bar=50 µm and the inset scale bar = 10 µm.
